# Covalent Binding of Dexamethasone to Polyimide Improves Biocompatibility of Neural Implantable Devices

**DOI:** 10.1002/adhm.202405004

**Published:** 2025-06-17

**Authors:** Giulia Turrin, Jose Crugeiras, Chiara Bisquoli, Davide Barboni, Martina Catani, Bruno Rodríguez‐Meana, Rita Boaretto, Michele Albicini, Stefano Caramori, Claudio Trapella, Thomas Stieglitz, Yara Baslan, Hanna Karlsson‐Fernberg, Fernanda L. Narvaez‐Chicaiza, Edoardo Marchini, Alberto Cavazzini, Ruben López‐Vales, Maria Asplund, Xavier Navarro, Stefano Carli

**Affiliations:** ^1^ Department of Environmental and Prevention Sciences University of Ferrara Via Luigi Borsari, 46 Ferrara 44121 Italy; ^2^ Institute of Neurosciences Department of Cell Biology Physiology and Immunology Universitat Autònoma de Barcelona Av. Can Domènech, ed M Bellaterra 08193 Spain; ^3^ Department of Chemical Pharmaceutical and Agricultural Sciences University of Ferrara Via Fossato di Mortara, 17 Ferrara 44121 Italy; ^4^ Centro de Investigación Biomédica en Red en Enfermedades Neurodegenerativas (CIBERNED) Instituto de Salud Carlos III Av. Monforte de Lemos, 3–5 Madrid 28029 Spain; ^5^ Laboratory for Biomedical Microtechnology Department of Microsystems Engineering‐IMTEK & BrainLinks‐BrainTools Center IMBIT//Neuroprobes University of Freiburg Georges‐Köhler‐Allee 201 79110 Freiburg Germany; ^6^ Department of Microtechnology and Nanoscience Chalmers University of Technology Kemivägen 9 Gothenburg 412 58 Sweden; ^7^ Council for agricultural research and economics – CREA via Manziana, 30 Rome 00184 Italy

**Keywords:** dexamethasone, drug delivery systems, foreign body reactions, intraneural electrodes, neuroprostheses, surface chemistry

## Abstract

Neural implants are widely used in prosthetic applications to interact with the peripheral nervous system, but their long‐term functionality is compromised by foreign body reactions (FBR). Thanks to its high biocompatibility, polyimide *poly* (biphenyl dianhydride)‐*p*‐phenylenediamine (BPDA‐PDA) represents a suitable material to fabricate ultrathin and ultra‐flexible neural implants. This study explores the surface functionalization of BPDA‐PDA, the electrically inert component of the neural implant. The novelty of this approach relies on the fact that dexamethasone (DEX covalently bound to BPDA‐PDA, enabling its sustained release over a period of at least 9 weeks. In vitro assays demonstrate that this strategy reduce the production of pro‐inflammatory markers in macrophages. In addition, the biocompatibility of the functionalized material has been ensured by evaluating the viability of dorsal root ganglia (DRG) neurons. Furthermore, in vivo implantation of DEX functionalized BPDA‐PDA substrates shows a significant reduction in inflammatory cell infiltration and fibrotic capsule thickness formed around the devices. These findings suggest that local release of DEX from the electrically inactive scaffold of neural implants may enhance their long‐term stability and performance by mitigating the FBR.

## Graphical abstract

The covalent binding of dexamethasone to the surface of polyimide represents the electrically inert part of implantable intraneural devices. This ensures a sustained release of the drug for 9 weeks. In vitro and in vivo tests confirm the reduction of the foreign body reactions and the improved biocompatibility of dexamethasone functionalized polyimide.

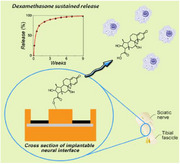

## 1 Introduction

A variety of active medical devices are used for treatment and rehabilitation strategies to modulate neuropathological alterations in nerve activity, and to replace or restore lost functions or organs and limbs.^[^ ^1^ ^]^ In the case of neuroprostheses, a technical system is interfaced with the subject's nervous system, with electrical interfaces being the most common. Cochlear implants and deep brain stimulation systems are examples of implantable systems in clinical use. Different types of electrodes have been designed for interfacing with the peripheral nervous system (PNS).^[^ ^2^ ^]^ These neural implants may be included as bidirectional interfaces for prosthetic applications for upper or lower limb amputees, in modulation of urinary bladder function, or in vagus nerve stimulation to modulate autonomic nervous system functions.^[^ ^1^, ^3^ ^]^ Intraneural electrodes, implanted either longitudinally or transversely in the peripheral nerves allow good bidirectional communication with the nervous system, i.e., they can be used to stimulate nerve fibers inducing motor actions or sensory feedback, and they also allow recording of nerve impulses conducted by nearby axons. However, their implantation results in the onset of the foreign body reaction (FBR), a phenomenon that, depending on the biocompatibility of the implant, ultimately compromises the functionality of the neural electrode over time.^[^ ^4^ ^]^

The FBR is the first response of the immune system against any implanted device and is characterized by an inflammatory phase triggered by macrophages and leukocytes, followed by a fibrotic phase in which fibroblasts form a fibrotic tissue around the implant. Material selection and device design influence the FBR of any implantable device. While general statements about cytotoxicity (surface biocompatibility) can be derived from in vitro studies on cells, influence with the target tissue in a well‐defined intended use (structural biocompatibility) needs in vivo experiments to come to sound conclusions. The international standard ISO 10993 “Biological evaluation of medical devices” delivers a set of rule‐based investigations to evaluate and assess the biocompatibility of any medical device, might it be extracorporeal or implantable. Implants in the PNS have been introduced since the 1960s starting with circumferential cuff electrodes and epineural wires manufactured with means of precision mechanics.^[^ ^2^ ^]^ Since the new millennium, flexible micromachined devices have been proposed.^[^ ^5^ ^]^ The polyimide biphenyl tetracarboxylic dianhydride‐paraphenylenediamine (BPDA‐PDA) has been chosen because of its lower than 1% water uptake, chemical resistivity, and low electric permeability.^[^ ^6^ ^]^ This material has shown high stability in chronic in vitro tests under accelerated aging, no cytotoxic effects in tests according to ISO 10993‐part 5, and devices showed good functionality in pre‐clinical studies in the peripheral and central nervous system.^[^ ^7^ ^]^ The approach of a flexible electrode being transversally or longitudinally implanted in peripheral nerves (TIME – transversal intrafascicular multichannel electrode; LIFE – longitudinal intrafascicular electrode) has been even transferred in clinical studies for sensory feedback after amputation of upper and lower limbs.^[^ ^8^ ^]^ Such implants showed high selectivity with stimulation thresholds in chemically safe stimulation levels and good stability over implantation periods of up to six months.^[^ ^9^ ^]^ However, despite good functionality in general, a significant FBR with a fibrotic capsule around the electrodes developed, which limits recording properties and increases stimulation thresholds over time.

Few studies have investigated factors influencing the FBR in the peripheral nervous system.^[^ ^4^, ^10^ ^]^ Recently, the extent of the inflammatory as well as the remodeling phases of the FBR to longitudinal implants in the peripheral nerve have been characterized, thus determining possible targets for reducing the encapsulation and improving electrode outcome.^[^ ^4^, ^11^ ^]^ Anti‐inflammatory drugs, such as dexamethasone (DEX) and ibuprofen, showed positive effects in reducing the thickness of the tissue capsule around intraneural devices by mitigating macrophage activation when systemically administered.^[^ ^12^ ^]^ Nevertheless, systemic administration of these drugs leads to secondary effects, making localized delivery of FBR modulatory agents at the site of the implant the most preferred strategy. The local release of DEX represents an interesting alternative to systemic administration because it permits both a significant reduction of the drug dosage and its direct delivery into the active site of the inflammation, namely the tissue intimately in contact with the neural implant. In general, this approach is oriented to the incorporation of the drug on the full surface of the electrode, to reduce the inflammatory reaction within the surrounding area in the nerve, thereby reducing the encapsulation and preserving their electrical functionality over time. In this context, the conductive polymer poly(3,4‐ethylenedioxythiophene), which is known as PEDOT, has been used since it can incorporate DEX or other active drugs, by electrostatic or covalent approach.^[^ ^13^ ^]^ However, in such cases, the drug is only present at the small active sites of the device, which represent a quite small area. The direct functionalization with the desired drug of the electrically passive surface of the implant (the BPDA‐PDA structure for instance) may represent an interesting alternative since this part of the neural device constitutes the largest area exposed to the tissue.^[^ ^13d^ ^]^ Thus, this approach may produce a more efficient reduction of the FBR, better than just adding the drug to the small conductive site area.

Recently, a hydrogel scaffold structure was covalently bound to polyimide‐coated Pt‐Ir wires and used as neural microelectrodes. This structure was subsequently loaded with DEX, which can be delivered through a diffusion‐controlled mechanism, considering that the drug is not covalently grafted on the polyimide film.^[^ ^14^ ^]^ In the present study, a covalently grafted drug delivery platform was built on the surface of BPDA‐PDA. Subsequently, the covalent incorporation of DEX on this platform was achieved through the formation of ester bonds, yielding a system that ensures sustained release of the drug. The polyimide BPDA‐PDA was selected considering its well‐known and demonstrated biocompatibility together with its use in long‐term implantable biomedical devices.^[^ ^6^, ^7^ ^]^ For simplicity, BPDA‐PDA will be abbreviated as PI.

## 2 Results and Discussion

### 2.1 Synthetic Strategy

The absence of any reactive functional group on PI makes this polymer extremely stable and inert toward chemical and biochemical reactions (see Figure S1a, Supporting Information). Thus, to introduce functional groups that can form covalent bonds with the desired substrate, like DEX, the chemical surface structure of PI must be activated. Several strategies can be considered to activate the surface of PI, which can be divided into dry or wet processes, respectively.^[^ ^15^ ^]^ Among wet processes, alkaline hydrolysis is one of the most common methods adopted to activate the polyimide surface. The chemistry of this protocol is well established and accounts for the formation of carboxylic acid groups that can be used to create ester or amide bonds to covalently graft the desired molecular substrate on the surface of PI. Typically, the PI is treated with a solution of 1–5 m KOH, while optimization of the temperature and the reaction time enables the control of the modified layer thickness. For instance, it was reported that the activation of PI in 1 m KOH at 50 °C for 10 min yielded a modification depth on the order of 210 Å.^[^ ^16^ ^]^ As expected, the progression of the KOH‐modified region to the inner part increases with the treatment time as well as the KOH concentration.^[^ ^17^ ^]^ As shown in Figure S1b (Supporting Information), the primary alcohol of DEX can be conveniently used to form ester linkages with carboxylic acid moieties. Thus, its subsequent release pathway is based on the hydrolysis of such an ester bond.^[^ ^18^ ^]^

**Figure 1:**
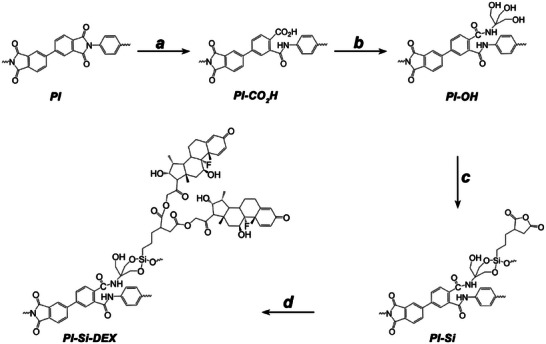
Schematic representation of the synthetic route to obtain PI covalently functionalized with DEX. a) KOH 5 m, 50 °C; 10 min; b) Trizma base, DCC, absolute ethanol, 50 °C, 24 h; c) TESPSA 4% sol in anhydrous toluene, r.t., 3 days; d) DEX, DCC; DMAP, ACN, 50 °C, 4 days.

In this study, the covalent attachment of DEX to PI was achieved through the formation of an ester linkage between DEX and the surface of PI, which was derivatized according to the synthetic route depicted in **Figure** **1**.

In the first step, the surface of PI was activated by means of KOH, which leads to the formation of pendant carboxylic acid moieties, to give the corresponding PI‐CO_2_H.^[^ ^19^ ^]^ At this stage, pendant hydroxyl groups were generated by a reaction of PI‐CO_2_H with the amino‐alcohol *tris*(hydroxymethyl) aminomethane, to give the intermediate PI‐OH. Hydroxyl groups were subsequently used to build a 3D vertically structured silane film, through the formation of strong covalent bonds. For this purpose, the commercially available triethoxysilylpropyl succinic anhydride (TESPSA) was chosen for several reasons. First, the presence of 3‐alkoxy‐silane units enables the formation of Si─O─C and Si─O─Si covalent bonds, yielding the construction of a siloxane film.^[^ ^20^ ^]^ Second, the succinic anhydride group can be strategically used to create ester bonds with the primary alcohol unit of DEX. In addition, the high biocompatibility of TESPSA coatings was reported in the literature, thereby supporting the choice of adopting this silane in this study.^[^ ^21^ ^]^ Thus, the resulting PI‐Si platform was used to incorporate DEX through the classic Steglich's esterification method, to give the final substrate PI‐Si‐DEX.

### 2.2 Surface Analysis

**Figure 2:**
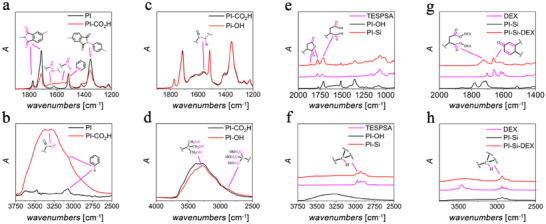
ATR‐FTIR spectral comparison: a, b) PI‐CO_2_H versus PI; c, d) PI‐OH versus PI‐CO_2_H; e, f) PI‐Si versus PI‐OH and TESPSA; g, h) PI‐Si‐DEX versus PI‐Si and DEX. A pink line links representative functional groups and their vibrational modes. In a) the absorbances are normalized (y‐axis) with respect to the signal at 1514 cm^−1^.

ATR‐FTIR analysis was performed in order to confirm the surface functionalization strategy reported in **Figure** **2** following each step.^[^ ^16^, ^22^ ^]^ As depicted in Figure 2a, the typical signatures of PI can be outlined by the presence of the strong band at 1711 cm^−1^ together with a less intense peak at 1772 cm^−1^, which are ascribed to the carbonyl vibrational mode of the imide ring. In addition, the C─N stretching mode of the imide functional group can be observed at 1353 cm^−1^.

**Figure 3:**
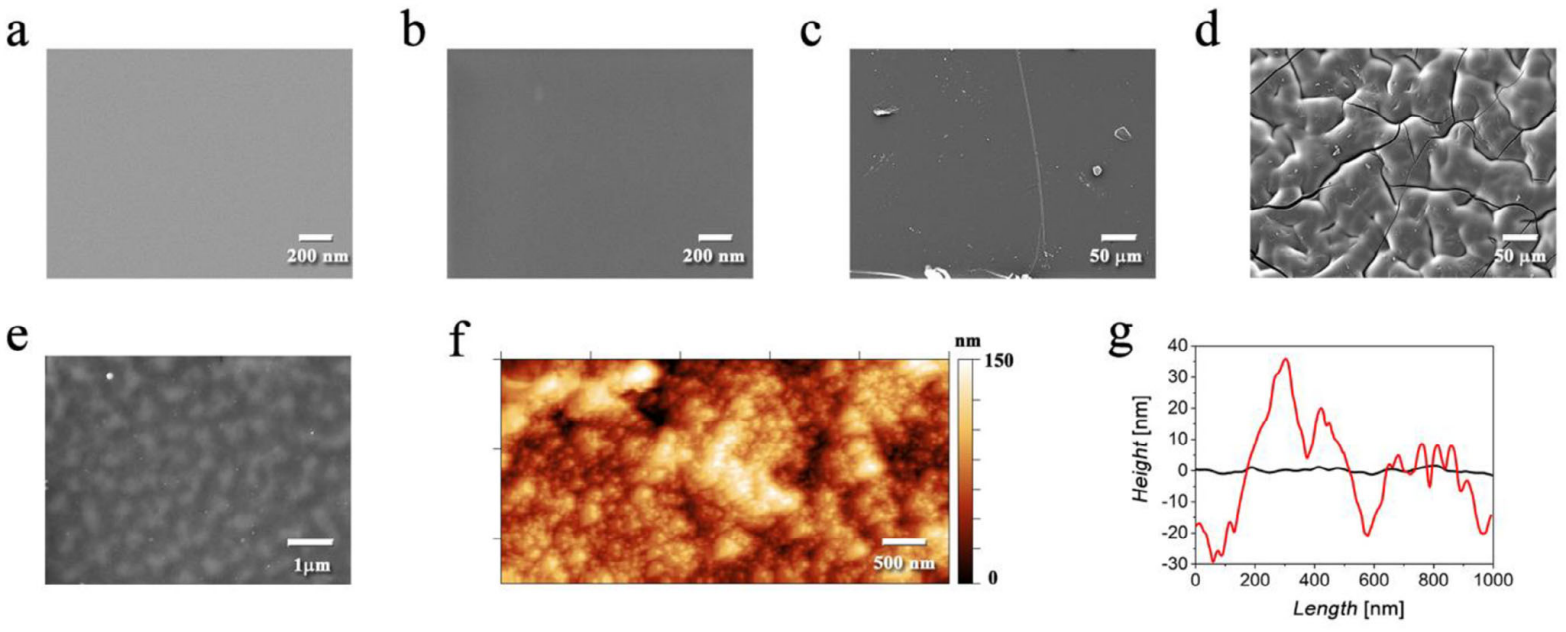
SEM characterization of a) PI, b) PI‐CO_2_H, c) PI‐Si, d, e) PI‐Si‐DEX; f) AFM map of PI‐Si‐DEX; g) representative cross‐sectional profiles of PI (black line) and PI‐Si‐DEX (red line).

The C═C vibrations for the BPDA and PDA aromatic rings can be observed at 1621 and 1514 cm^−1^. The alkaline hydrolysis of the surface imide groups of PI is expected to translate into a reduction of the absorbance for the signal at 1710 cm^−1^, according to Lambert Beer's law. Nevertheless, under these conditions, the aromatic rings are chemically stable, and thus, the absorbance of the peak at ≈1514 cm^−1^ is expected to remain constant after the activation step. Thus, the parameter A_1711_/A_1514_, which reflects the ratio between the absorbance at 1711 and 1514 cm^−1^, respectively, was used to monitor the progression of the activation step. In **Figure** **3**a, the ATR‐FTIR spectra of PI and PI‐CO_2_H are reported, and their relative intensities were normalized with respect to the signal at 1514 cm^−1^. It can be easily seen that upon partial hydrolysis the intensity of the distinctive imide ring signals at 1722, 1711 and 1353 cm^−1^ clearly decreases, as expected, being a consequence of a surface partial depletion of these functional groups. In particular, pristine PI exhibited an A_1711_/A_1514_ value of ≈2.6, whereas this parameter drops to ≈1.2 after KOH treatment, which is consistent with a reduction of the intensity of the signal at 1711 cm^−1^ (see Figure 2a) as a consequence of the partial hydrolysis of surface imide sites. Furthermore, the alkaline‐treated PI‐CO_2_H exhibits two new signals at 1650 and 1559 cm^−1^ which are linked to the newly generated amide and carboxylate functions, respectively, as summarized in Figure 1.^[^ ^23^ ^]^ The formation of carboxylic groups is also corroborated by the appearance of the broadband in the spectral range of 3000–3500 cm^−1^, as shown in Figure 2b. In this region, PI exhibited only a weak peak at ≈3067 cm^−1^ which can be ascribed to the aromatic C─H stretching vibrations. This signal can be still observed for PI‐CO_2_H as a shoulder of the more prominent broad peak centered at ≈3300 cm^−1^. The confirmation that the alkaline treatment is limited to the uppermost layers of PI is evidenced by the fact that the intensities of the main peaks for the imide structure at 1772 and 1711 cm^−1^ were reduced but not completely eliminated (see Figure 2a). It was reported that there is a linear relationship between the thickness of PI and the intensities of the imide signals. Therefore, the penetration depth value for the KOH treatment can be estimated by ATR‐FTIR analysis according to the thickness‐absorbance linear relationship *A*∝*t* observed by Lee et al. for PI samples.^[^ ^16^, ^22^ ^]^ A reduction of the absorbance of the imide group is expected after KOH surface hydrolysis and the extent of this reduction to be proportional to the depth of the hydrolytic treatment. In the case of pristine PI, the thickness of unmodified polyimide *t_PI_* corresponds to the evanescent wave penetration depth *d_p_* (see Equation (5)) of the ATR beam, that is *d_p_* = *t_PI_* . Specifically, the absorbance of imide signals (at 1770 and 1711 cm^−1^) is larger for PI if compared to PI‐CO_2_H, because in the latter case a certain amount of imide surface sites is hydrolysed by KOH treatment. When ATR analysis is performed on PI‐CO_2_H the parameter *d_p_* can be expressed by the Equation (1):

(1) $$d_{p}=t_{PI^{\prime }}+t_{CO_{2}H}$$

where *t*_*PI*′_ and $t_{CO_{2}H}$ are the thicknesses of the unmodified and modified polyimide layers, respectively. Thus, the depth of KOH treatment, which corresponds to the thickness of carboxylated surface sites $t_{CO_{2}H}$ in PI‐CO_2_H, can be established according to Equations (2) and (3):

(2) $$t_{CO_{2}H}=d_{p}\;-t_{PI^{\prime }}$$

(3) $$t_{PI^{\prime }}=\left(A_{PI^{\prime }}/A_{PI}\right)t_{PI}$$

where *A*_*PI*′_ and *A_PI_* are the absorbance values observed for modified PI‐CO_2_H or pristine PI, respectively, at the selected wavenumbers of 1770 or 1711 cm^−1^, which are referred to as the signals of the imide ring. Following this approach, a penetration depth of ≈100 nm is estimated for 5 m KOH treatment, which converts PI to the activated PI‐CO_2_H. A comparable value was obtained in an analogous manner by adopting the FT‐IR analysis in transmission mode, as reported in Table S1 (Supporting Information). It is important to note that the mean thickness value for PI‐CO_2_H was not significantly different than that of PI (see data in Table S2, Supporting Information), which clearly indicates that the KOH treatment yielded surface carboxylic acid groups that are stably anchored to the underlying native PI. In other words, the basic treatment did not produce surface erosion which should be linked to a thickness reduction of the treated PI with respect to the pristine one. In addition, considering that the thickness value of the PI substrates in this study was ≈12 µm, this clearly means that the KOH activation of PI was confined to the uppermost layers, representing ≈1% of the overall film depth.

In the subsequent synthetic step, ATR‐FTIR analysis of the intermediate PI‐OH outlined a stronger signal at 1560 cm^−1^, if compared to the spectra of PI‐CO_2_H, which is due to the coupling of N─H bending and C─N stretching of new secondary amide sites in PI‐OH (Figure 2c). In addition, a further slight reduction of the signals at 1772, 1711 and 1353 cm^−1^ can be ascribed to the imide ring opening promoted by nucleophilic attack of the amino groups of Trizma. The new symmetric N─H stretching can be observed by the increased secondary peak at ≈3260 cm^−1^, whereas the C─H vibrations of Trizma yielded the formation of two new shoulders at 2964 and 2894 cm^−1^, as displayed in Figure 2d.^[^ ^24^ ^]^ The construction of TESPSA film on PI was achieved by treating PI‐OH samples in a toluene solution of the silane precursor.^[^ ^20b^ ^]^ In these conditions, the silanization occurs through the condensation between the alkoxy groups of TESPSA and the surface hydroxyl groups of PI‐OH.^[^ ^25^ ^]^ Nevertheless, the likely availability of surface water is expected to favor multilayer silanization reactions, thereby leading to the formation of the 3D‐structured TESPSA film.^[^ ^26^ ^]^ The presence of the silane coating in PI‐Si can be clearly confirmed by means of ATR‐FTIR analysis.^[^ ^27^ ^]^ As depicted in Figure 2e, the ATR‐FTIR spectra of PI‐Si are mostly dominated by the distinctive features of the silane precursor TESPSA. In particular, the signals at 1780 and 1860 cm^−1^ are linked to the symmetric and asymmetric stretching vibrations of carbonyl groups in the succinic anhydride ring of TESPSA units. The strong peak at 1716 cm^−1^ refers to the carboxylic groups generated by partial hydrolysis of the anhydride ring during the silanization protocol. Besides, the peaks at 1514 and 1353 cm^−1^ are ascribed to the underlying PI‐OH, as discussed above, together with the shoulder peak at 1711 cm^−1^. In addition, the strong signal at ≈1069 cm^−1^ is linked to the silanol Si‐OH/siloxane Si‐O‐C/Si‐O‐Si groups, whereas three intense signals at 2980, 2933, and 2879 cm^−1^ can be clearly assigned to the C─H stretching modes of TESPSA propylic chains, as reported in Figure 2f. The last step of the overall synthetic pathway is represented by the incorporation of DEX through the formation of surface covalent bonds. The most prominent ATR‐FTIR signatures that confirm the presence of DEX covalently attached are the new strong signals at 1663 and 1721 cm^−1^, as shown in Figure 2g. The former peak is linked to the *α‐β* double bond framework conjugated with ─C═O systems whereas the latter one is indicative of the formation of an ester linkage between the hydroxyl group of DEX and the carboxylic function of TESPSA.^[^ ^18b^ ^]^ In addition, with respect to the original PI‐Si, the succinic anhydride peaks at 1780 and 1860 cm^−1^ were not detected in PI‐Si‐DEX. This is indicative of a high degree of DEX functionalization that occurs through the opening of the succinic anhydride moiety, which clearly supports the main goal of DEX functionalization through the formation of an ester linkage.^[^ ^18b,c^ ^]^ Finally, the appearance of a broad band centered at ≈3400 cm^−1^ and the increased intensity of the signal at 2936 cm^−1^ further corroborate the presence of DEX on the surface of PI‐Si‐DEX, taking into account that this signal can be linked to the OH and C─H groups of DEX, as displayed in Figure 2h.

Contact angle measurements were also collected to monitor the change in wettability of PI‐based samples after the various treatments (see Table S3, Supporting Information). It was found that the surface becomes more hydrophilic upon KOH activation, as the initial contact angle of ≈66° observed for PI decreased to ≈53° for PI‐CO_2_H. After this initial step, the introduction of hydroxyl functions on PI‐OH, the subsequent construction of the silane film of PI‐Si as well as its functionalization with DEX to Pi‐Si‐DEX, yielded no substantial variations in wettability if compared to PI‐CO_2_H and these samples exhibited a contact angle value of ≈50°–60°.

SEM analysis of PI outlined an extremely smooth surface structure, which is apparently featureless down to the sub‐micrometer scale, as can be seen in Figure 3a. This characteristic was preserved even after the strong alkaline treatment to give the intermediate PI‐CO_2_H, as depicted in Figure 3b. The construction of PI‐Si produced the formation of a compact layer of silane film on the top of the PI‐OH surface (see Figure 3c).

Interestingly, the final incorporation of DEX resulted in a dramatic change in the surface morphology of PI‐Si‐DEX, if compared to the original PI‐Si substrate. In the sub‐millimeter domain, PI‐Si‐DEX exhibited a globular structure, with a globule's average width of ≈100 µm, as displayed in Figure 3d. These globules are separated by cracks that are distributed randomly over the surface of PI‐Si‐DEX. Nevertheless, at closer inspection, smaller globules can be observed on the surface of PI‐Si‐DEX as well, as shown in Figure 3e. AFM analysis confirmed a significantly larger surface roughness for PI‐Si‐DEX (R_a_ ≈ 21 nm) if compared to the original PI (R_a_ ≈ 0.5 nm), on the order of 26 and 0.6 nm, respectively (see data in Table S4, Supporting Information). In addition, AFM analysis provided a deeper understanding of the nature of PI‐Si‐DEX nanostructure. Indeed, the big globules outlined by SEM analysis are likely to result from the aggregation of smaller ones, with a grain size on the order of ≈ 50 nm, as can be seen in Figure 3f,g. The largest vertical distance R_max_ between the highest and lowest points of the profile (within the evaluation area of 5×2.5 µm^2^) was found on the order of 185 nm (see Table S4, Supporting Information). As reported in Figure S2 (Supporting Information), cross‐sectional SEM analysis of a sample of PI‐Si‐DEX confirmed a thickness value that ranges between 11.8 and 12 µm, which suggests that the thickness of the silane film ranges between 100 and 200 nm, according to AFM analysis.

**Figure 4:**
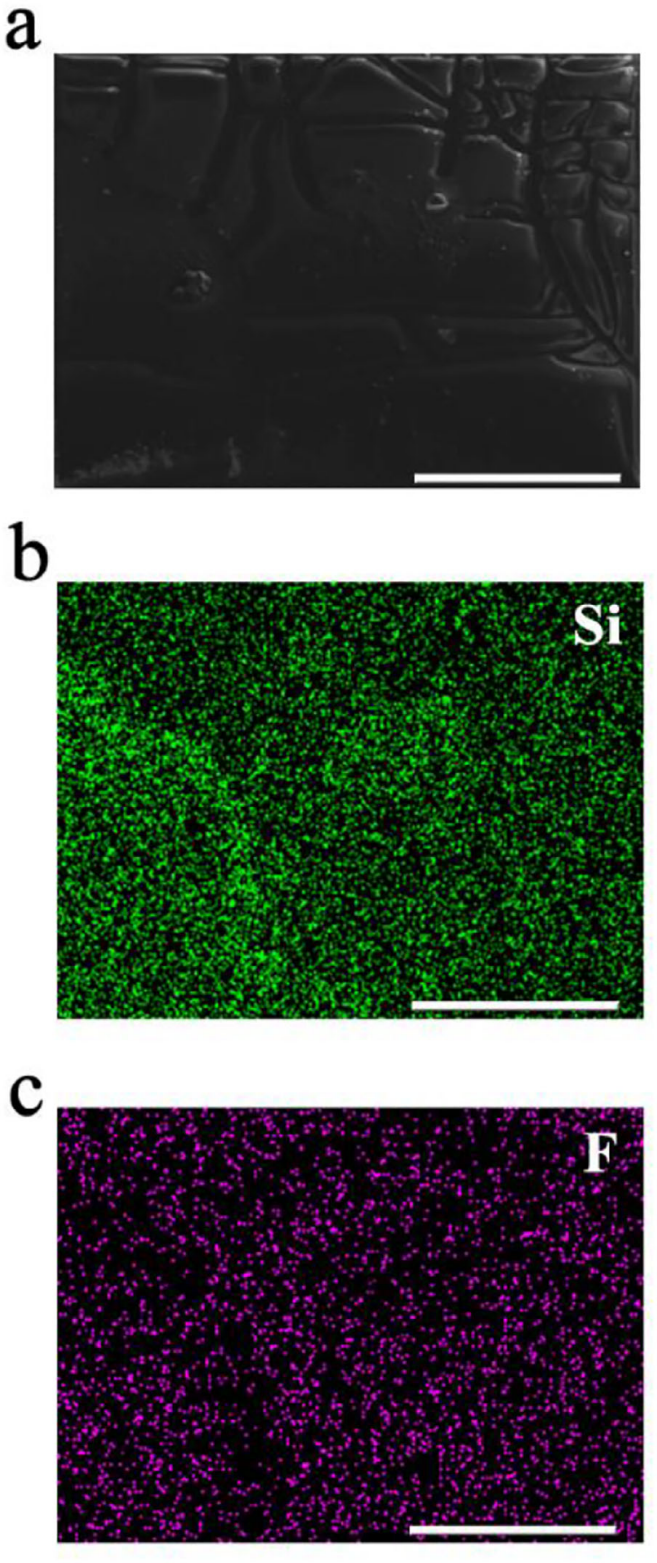
SEM/EDX elemental mapping of PI‐Si‐DEX: a) SEM image, b, c) Si and F atoms distribution, respectively. Scale bars are 250 µm.

As shown in **Figure** **4**, an SEM/EDX map analysis outlined how F atoms are well dispersed along with Si atoms to indicate homogeneous incorporation of DEX over the surface, which is beneficial to maximize drug loading per surface area. Furthermore, considering that the only contribution of F atoms derives from the DEX source, according to its chemical structure (see Figure S1b, Supporting Information), the experimental F/Si atomic ratio for PI‐Si‐DEX can provide the DEX‐TESPSA esterification stoichiometry. Indeed, SEM/EDX elemental analysis confirmed a F/Si atomic ratio value of ≈2 (1.9 ± 0.3, *n* = 8) which is consistent with a high degree of esterification of the carboxylic acid functions of TESPSA by DEX.

### 2.3 DEX Release Analysis

**Figure 5:**
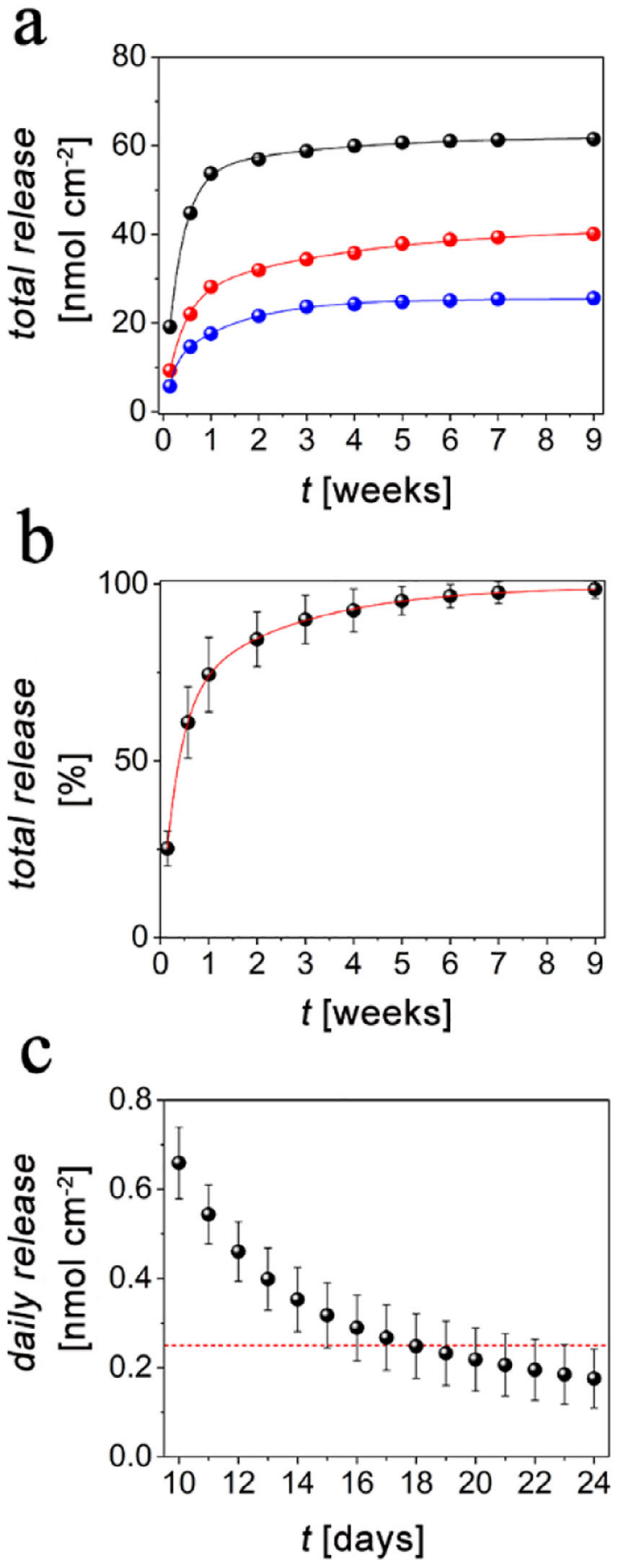
Release profiles of DEX from three different PI‐Si‐DEX substrates at pH 7.4: a) experimental data (dots) were fitted according to the bi‐exponential model (lines) cumulative release for three PI‐Si‐DEX substrates (black, blue, and red line/dots are referred to the three different samples, respectively); b) mean cumulative release as a percentage of total released DEX (this parameter was extracted by the fitting according to Equation (4), see Table S8, Supporting Information); c) daily release data obtained from the fitting of experimental data, see also Table S9 (Supporting Information), showing the time window during which the amount of released DEX is ≥ 0.25 nmol cm^−2^ (dashed red line). In (b) experimental data are dots, while the line refers to the fitting trace; in (b, c) data are reported as mean ± SD (*n* = 3).

The in vitro release profiles of DEX from three PI‐Si‐DEX samples were monitored over a total time of 9 weeks. This time interval was chosen to go beyond the initial inflammation period that is activated immediately after the probe insertion, and which lasts typically 3–4 weeks.^[^ ^4a^ ^]^ As depicted in **Figure** **5**a (see also Tables S5 and S6, Supporting Information for experimental release data), the DEX release profile outlines a burst phase during the first two weeks followed by a slower drug release rate, which lasted until the end of the assay. An average total amount of DEX of 51 ± 15 nmol cm^−2^ was released from the PI‐Si‐DEX‐based drug delivery platform. To gain further insights into the kinetics and mechanism of DEX release from PI‐Si‐DEX, experimental release data were fitted to various kinetic models. In particular, the mathematical models for this study are the zero order, first order, Higuchi, Korsmeyer‐Pappas models, Weibull, and a biexponential‐biphasic kinetic function.^[^ ^28^ ^]^ The goodness of the fitting models was estimated by comparing their corresponding correlation coefficient (*R^2^*). As reported in Table S7 (Supporting Information), the biexponential Equation (4) provided the best‐fitting result:

(4) $$D_{t}=\left(Ae^{-k_{1}t}+Be^{-k_{2}t}\right)+D_{Total}$$

In Equation (4) *D_t_* is the quantity of DEX released in each incubation period, *D_Total_* is the total amount of DEX that can be delivered by PI‐Si‐DEX, *k*_1_ and *k*_2_ are rate constants that characterize both release phases, while *A* and *B* have their coefficients (see Table S8, Supporting Information).^[^ ^29^ ^]^ The goodness of the bi‐exponential model is corroborated by the presence of two distinct release phases as shown in Figure 5a. A rapid release phase can be distinguished during the first 1–2 weeks and, after this initial period, the release rate becomes slower, and this second phase lasted until the end of the assay. In addition, these two distinct phases of kinetic release can be explained by observing that the drug delivery occurs through the hydrolysis of the ester linkages between DEX and TESPSA groups. These active sites are distributed over the Si‐based platform and therefore it is likely that the outermost layers, put in direct contact with the electrolyte, deliver DEX at a faster rate if compared to the innermost regions of the porous scaffold. It is interesting to note that the faster component of the release kinetics exhibited a larger variability in amplitude among the analyzed samples, as can be deduced by the larger error bars during the first week, in Figure 5b. This may be related to the total amount of DEX that has been incorporated in PI‐Si‐DEX. Indeed, according to the fitting data reported in Table S8 (Supporting Information), both pre‐exponential factor *A* and kinetic constant *k_1_* scale with the total DEX quantity. By contrast, the slower components of the release kinetics (B) have generally a lower weight (from 1/3 to 1/5 compared to A), and are quite comparable in all samples, being insensitive to the total amount of the incorporated drug. This points to its physical origin in the residual fraction of DEX, which is either strongly retained by the surface or less accessible to hydrolytic cleavage by the electrolyte.

As can be observed in Figure 5, PI‐Si‐DEX can release DEX for almost two months, ensuring a higher dosage of the drug during the first ≈20 days. It is important to note that to significantly reduce immune‐mediated inflammation, a therapeutically relevant dosage of DEX should be locally delivered. From this perspective, it was reported that a release of 0.25 nmol cm^−2^ (0.1 µg cm^−2^) of DEX, which should correspond to a concentration of 0.2 µm within a 500 µm radius from the neural implant, may represent a therapeutically bioactive dosage of locally delivered DEX.^[^ ^30^ ^]^ Thus, the theoretical daily release of DEX was extracted from the fitting of experimental data shown in Figure 5a, according to the Equation (4). As displayed in Figure 5c, and detailed in Table S9 (Supporting Information) the daily dosage of DEX that can be delivered from PI‐Si‐DEX is above the ideal limit of 0.25 nmol cm^−2^ for an average period of ≈3 weeks.^[^ ^30^ ^]^ This particular release pathway may be beneficial for the construction of a stable chronically implanted device since it is well known that the reduction of the inflammatory response during the first 2–3 weeks after the insertion of the neural implant is highly effective.^[^ ^12^ ^]^

In addition, the PI‐Si‐DEX delivered locally an overall amount of DEX of 51 ± 15 nmol cm^−2^, which corresponds to 8–23 µg cm^−2^ of the drug. A healthy human body produces 5–28 mg d^−1^ of endogenous cortisol.^[^ ^31^ ^]^ The highest daily DEX release of ≈18 nmol cm^−2^ was found on the first day, which reflects a single local dosage of ≈7 µg cm^−2^ d^−1^. This would correspond to a release of 175 µg cm^−2^ d^−1^ of cortisol, considering that the potency of DEX is 25 times that of cortisol.^[^ ^32^ ^]^ Thus, considering that the total area of a TIME or LIFE intraneural electrode for humans is on the order of 2 mm^2^, this implant would release the highest dosage of DEX in the range of 160–460 nmol at day 1, which corresponds to 4–115 µg of cortisol, a thousand time less than the normal endogenous steroid production.

The stability of PI‐Si‐DEX was monitored during an overall period of 4 months, as detailed in the Experimental Section. SEM/EDX elemental analysis for the aged sample of PI‐Si‐DEX is reported in Figure S3 (Supporting Information). After this period, some areas of the sample are not covered by the silane film, which is consistent with partial and progressive hydrolysis of PI─O─Si bonds.^[^ ^25^ ^]^ Nevertheless, it can be clearly outlined that large areas of the sample are still covered by the silane film, but two different surface morphologies are present. Surprisingly, fluorine atoms can be detected even after 4 months, to indicate the presence of DEX, even though its quantification by HPLC becomes impossible when its concentration falls below the LOD. It is expected that the silane film is hydrolytically removed during the exposure to in vivo physiological environment, but the analysis of release confirms that this scenario may become relevant during a time window that is significantly longer than the DEX release pathway which is, as demonstrated above, well aligned with the 3–4 weeks needed to suppress or reduce the initial inflammation after the probe insertion.

### 2.4 Mechanical Analysis

**Figure 6:**
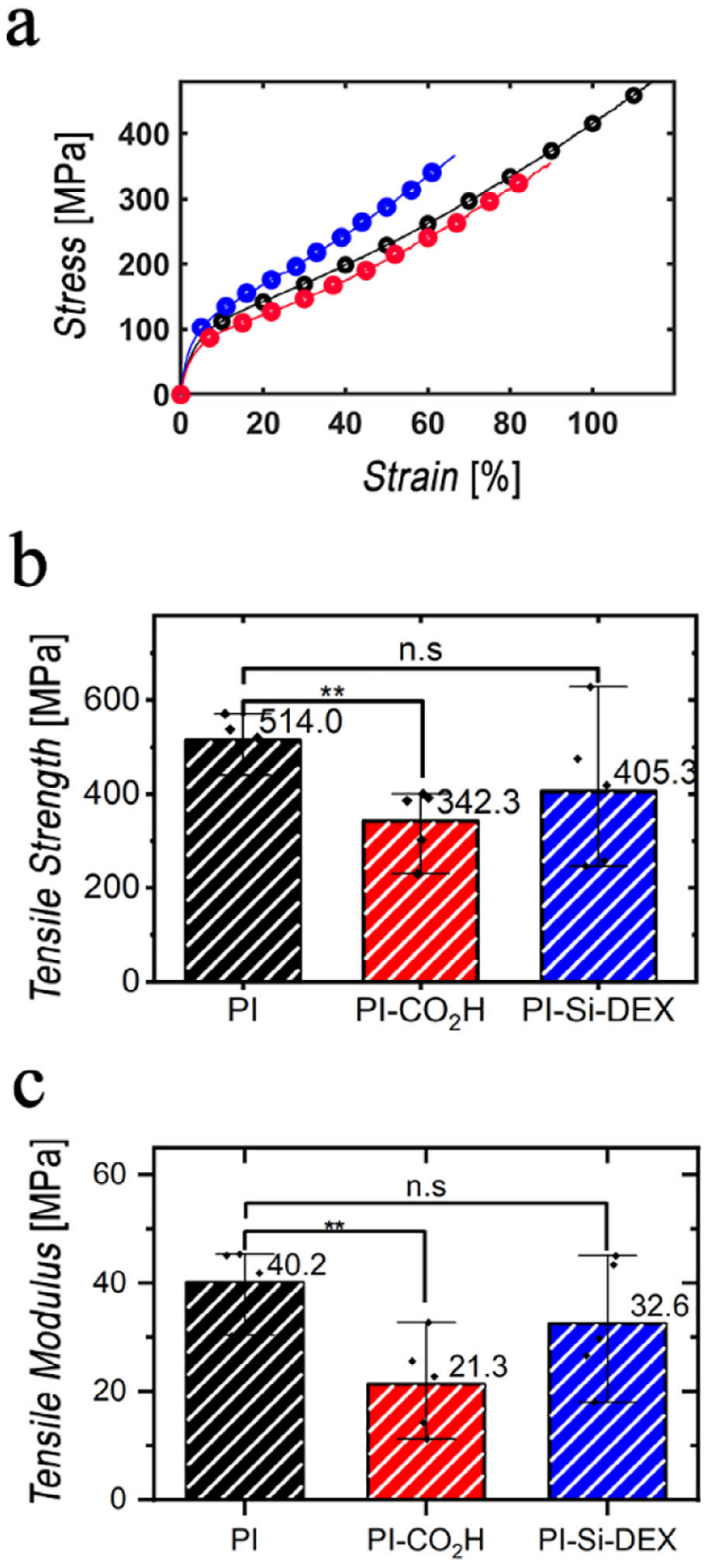
Tensile Testing Results for PI‐CO_2_ and PI‐Si‐DEX treatments. a) Stress–strain curves for PI (black), PI‐CO_2_H (red), and PI‐Si‐DEX (blue) samples; b) tensile strength at break, measured across *n* = 5 specimens for each treatment group; c) tensile modulus comparison between untreated and treated PI specimens. One‐way ANOVA test on *n* = 5 specimens for each treatment group. ^*^ *p* < 0.05, ^**^ *p* < 0.001, ^****^ *p* < 0.0001. Data are reported as mean ± SD.

Any functionalization method chemically changing the PI comes at the risk of other fundamental properties of the material changing as well. This is especially important to consider in the case of thin‐film layers where the proportional contribution of surface to the overall properties of the entire device will be substantial. To investigate if the functionalization had any major impact on the mechanical qualities of the film, we performed mechanical tensile tests. **Figure** **6** presents the tensile test results for PI‐CO_2_H and PI‐Si‐DEX substrates, compared to untreated PI controls. The stress–strain curves in Figure 6a reveal significant changes in mechanical behavior for Pi‐Si‐DEX. Figure 6b shows for PI‐CO_2_H a 33% reduction in tensile strength on average, likely due to KOH treatment which caused surface structural modifications. Additionally, the tensile modulus (Figure 6c), calculated during the elastic deformation phase, decreased by 46% after KOH activation in PI‐CO_2_H, indicating increased elasticity. This can be explained by considering that the KOH treatment of PI to give PI‐CO_2_H yields the controlled hydrolysis of surface imide chemical bonds. Going more in‐depth, the cleavage of surface imide C─N bonds is expected to yield a reduction of the tensile strength and modulus. In contrast, a slight increase in both tensile modulus (Figure 6b) and tensile strength (Figure 6c) was observed for PI‐Si‐DEX, if compared to PI‐CO_2_H, which is consistent with an overall enhancement in tensile strength and a slightly more rigid material. The modest increase in the tensile modulus of PI‐Si‐DEX reflects the reinforcement effect attained by the formation of covalent chemical bonds between the silane layer and the hydroxyl groups of PI‐OH surface, corroborating the covalent scenario regarding the attachment of the silane layer on PI surface. In addition, intermolecular Si—O—Si cross‐linkage occurring inside the silane layer is expected to further reinforce the building structure of PI‐Si‐DEX.

### 2.5 Effects on the Inflammatory Profile of Macrophages

Bone marrow‐derived macrophages are widely used to study macrophage activation in vitro, and the inflammatory profile of these cells in the presence of different agents. Following previous studies, we induced a pro‐inflammatory profile by stimulating macrophages with lipopolysaccharide (LPS).^[^ ^33^ ^]^

**Figure 7:**
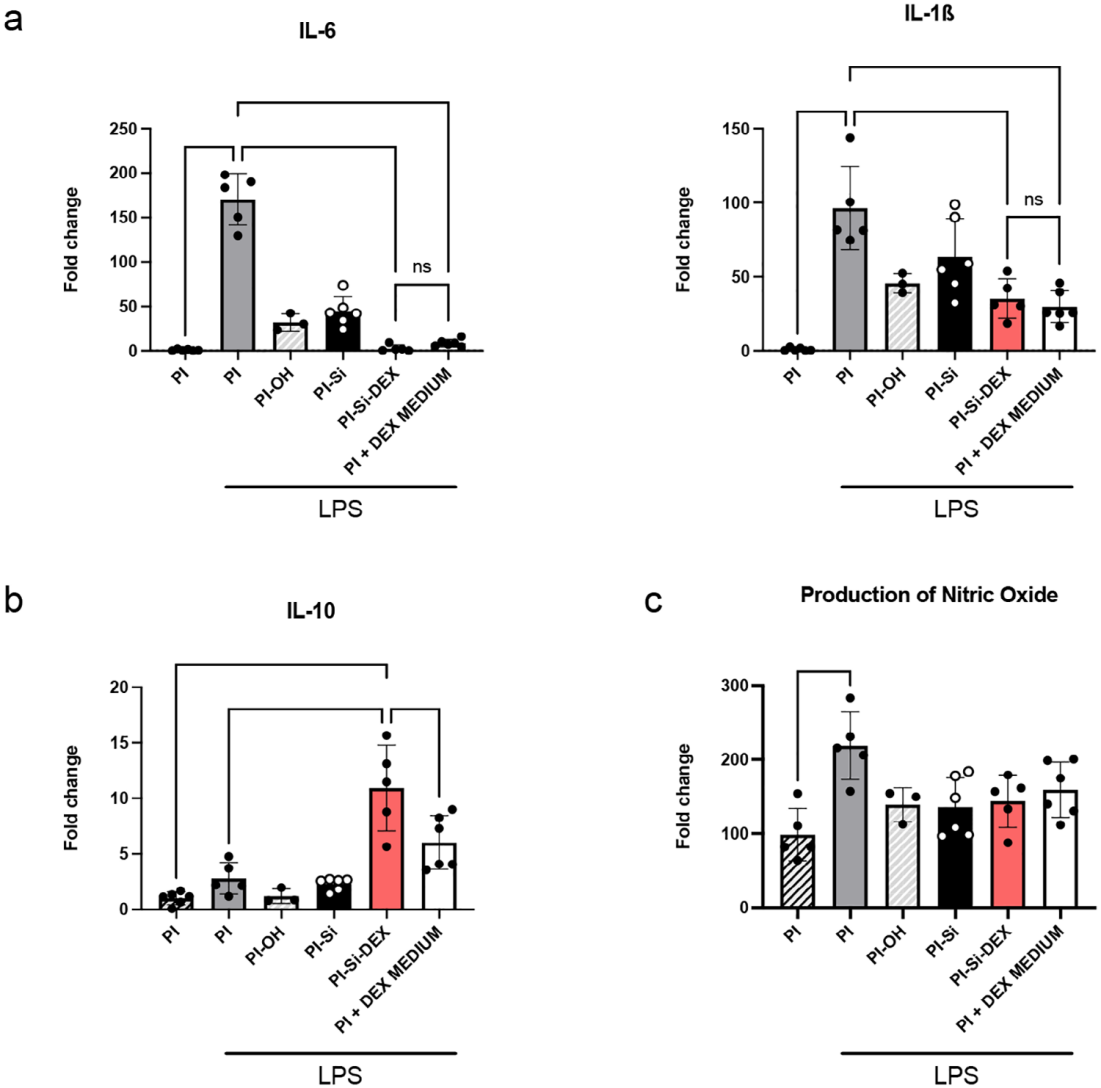
Inflammatory profile after LPS treatment in macrophages cultured on different PI conditions: a) expression levels of pro‐inflammatory cytokines IL‐6 and IL‐1ß; b) expression levels of anti‐inflammatory cytokine IL‐10; c) production of nitric oxide. *n* = 3–6 independent cultures. Kruskal–Wallis test for multiple comparisons. ^**^ *p* < 0.01, ^***^ *p* < 0.001, ^****^ *p* < 0.0001. The data are expressed as the fold change versus the control condition and are presented as the mean ± SD.

Regarding the gene expression of two pro‐inflammatory cytokines, IL‐6 and IL‐1ß, there was a substantial increase in the expression of both cytokines in the cultures treated with LPS compared to the control PI culture (**Figure** **7**a). In contrast, in the wells where DEX was present, either added to the medium (PI + DEX medium) or bound to PI (PI‐Si‐DEX), there was a significant decrease in the gene expression of both cytokines. On the other hand, the gene expression for anti‐inflammatory cytokine IL‐10 (Figure 7b) increased significantly in the case of PI‐Si‐DEX compared to the control as well as PI + LPS, indicating that PI‐Si‐DEX attenuates the pro‐inflammatory phenotype in the activated macrophages.

Measurement of nitric oxide (NO) production (Figure 7c) was used as an indicator of the inflammatory burst of the macrophages activated by LPS.^[^ ^34^ ^]^ While there is a notable, although not significant, reduction in NO production in the PI‐Si‐DEX condition compared to the PI + LPS, likely due in part to the low number of samples, the intermediate preparations of PI‐OH and PI‐Si have minimal effect. Interestingly, the reduction in NO production was more marked when DEX was bound to the PI substrate than when it was added to the medium as a solution. Macrophages in a pro‐inflammatory state produce greater amounts of extracellular NO, whereas treating murine macrophages in vitro with anti‐inflammatory agents decreases the release of NO.^[^ ^35^ ^]^

### 2.6 Effects on DRG Neurons

**Figure 8:**
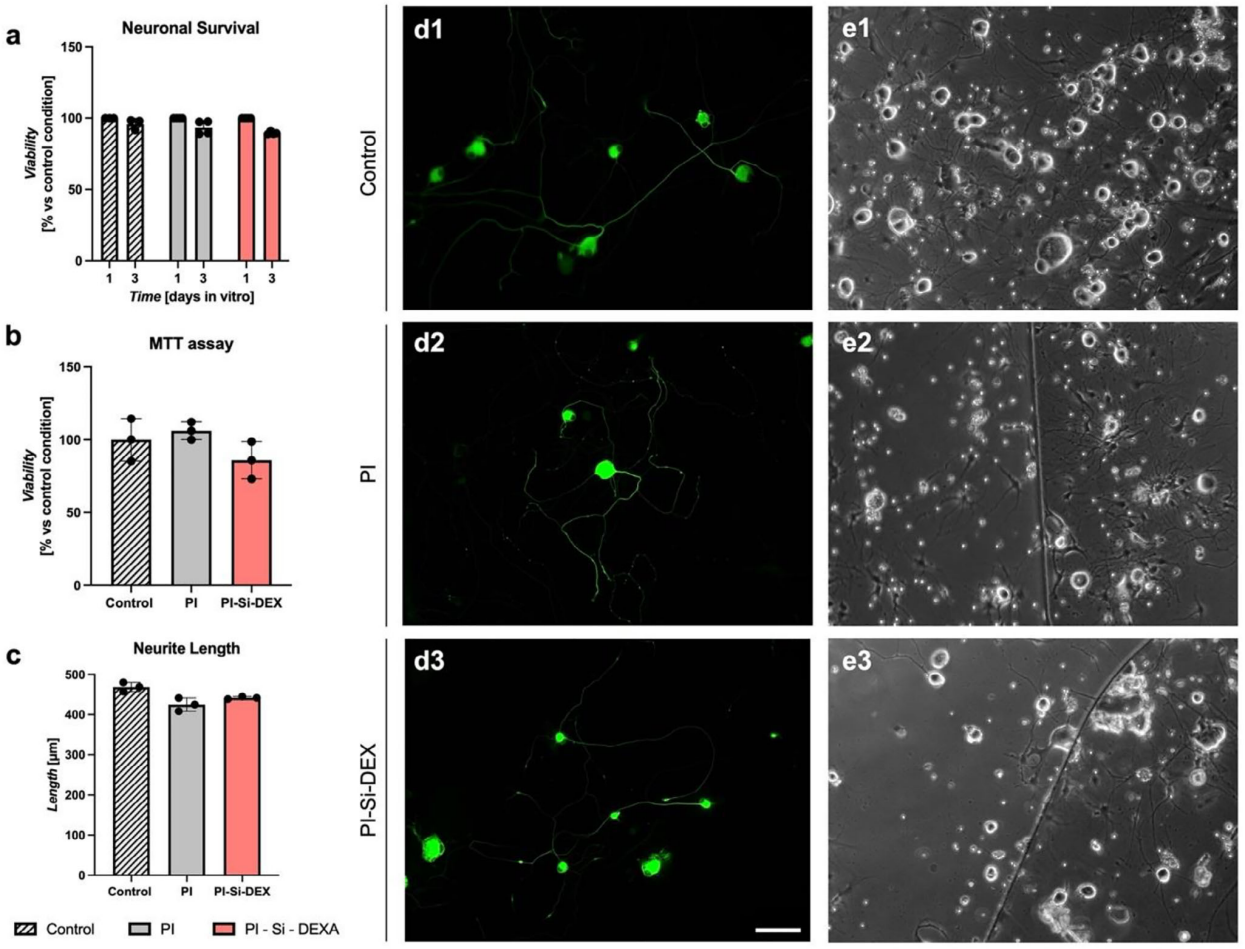
Effects on neuronal survival and neurite length in DRG culture. a) Neuronal survival after exposure to PI or PI‐Si‐DEX devices. b) MTT assay in the presence of PI and PI‐Si‐DEX. c) Measure of the neurite length in neurons plated with PI and PI‐Si‐DEX. Data for neuronal viability and MTT are expressed as fold change versus the control condition. Data are presented as mean ± SD. The Kruskal–Wallis test for multiple comparisons did not show any significant difference. d1–d3) Images of immunofluorescence against ß‐III‐tubuline used for neurite length measurements in a control well, a well with PI attached and a well with PI‐Si‐DEX attached, respectively; e1–e3) Microscope images of neurons plated in wells with the same conditions. Scale bar: 100 µm.

Before proceeding to in vivo studies, we analyzed in vitro the biocompatibility of the new bound PI devices using dorsal root ganglia (DRG) neurons, as in previous studies.^[^ ^7^, ^36^ ^]^ **Figure** **8** shows the effects of the presence of the PI devices, either PI alone or PI‐Si‐DEX on neuronal viability, measured by counting the number of neurons present in the wells (Figure 8a,e) or by the 3‐(4,5‐dimethylthiazol‐2‐yl)‐2,5‐diphenyltetrazolium bromide assay (MTT) (Figure 8b). There were no significant differences between conditions in either of the two tests, indicating that the PI devices tested do not impact neuronal survival. Figure 8d,e show images of neurons in culture in the presence of PI devices. In addition, the cultured neurons extended neurites of similar length on PI devices as in control cultures (Figure 8c,d). These results demonstrate that the PI‐Si‐DEX devices are biocompatible with DRG neurons, as evidenced by the MTT assay and neuronal counts, which show no significant differences compared to the control culture without PI. The arborization of cultured neurons in the presence of different substrates has been extensively studied, highlighting the importance of the substrate used or the presence of foreign agents, as they can potentially compromise neurite growth.^[^ ^37^ ^]^

### 2.7 In Vivo Assay to Reduce the FBR

Following the PI device implantation in the tibial nerve of the rat, two key features of the FBR were evaluated: the infiltration of inflammatory cells and the thickness of the tissue capsule formed around the device at two key time points, 2 and 8 weeks after implantation. The study was conducted at these two time points, because the peak of the macrophage infiltration occurs at 2 weeks, followed by a reduction in the inflammatory response observed at 8 weeks. Additionally, the tissue capsule around the devices acquires maximal thickness also at 2 weeks and is remodeled subsequently becoming stable at 8 weeks.^[^ ^37^ ^]^

**Figure 9:**
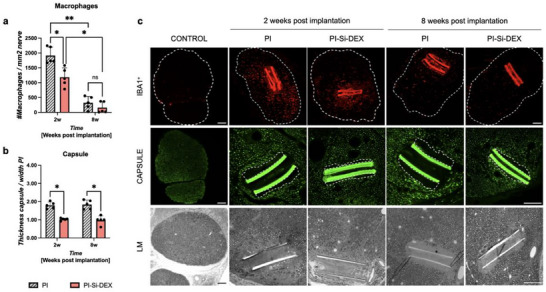
In vivo, FBR to intraneural PI control and PI loaded with dexamethasone devices. a) Histogram of the density of macrophages (Iba1+ cells) in the tibial nerve (delimited by a dotted line in the microphotographs) of animals implanted with a longitudinal intraneural PI control device or PI device with DEX at 2 and 8 weeks after the surgery. b) Histogram of the capsule thickness (dotted line in the capsule microphotographs) around the devices in the tibial nerve of animals implanted with PI control or PI loaded with DEX devices. Data are reported as mean ± SD; *n* = 5 per group. Two‐way ANOVA followed by Bonferroni post‐hoc test. ^*^ *p* < 0.05, ^**^ *p* < 0.001. c) The right panels show representative micrographs immunolabeled against Iba1 (red) and neurofilaments (green) and under light microscopy (LM). Note the high autofluorescence of the PI strips. The bottom row shows representative images taken at light microscopy showing a section of the nerves with the implanted device. Note the connective capsule around the PI devices and the lack of axonal damage. Scale bar: 100 µm.

The number of ionized calcium binding adaptor molecule 1 Iba1+ cells was significantly reduced in the case of PI‐Si‐DEX implants compared to the control untreated PI (**Figure** **9**a), suggesting that the locally released DEX from the implant diffused within the endoneurium, thereby reducing the recruitment of infiltrating macrophages at 8 weeks, as expected.^[^ ^37^ ^]^ the number of macrophages was largely reduced in all the nerves implanted, without significant differences between the two groups. Additionally, both at 2 and 8 weeks, the area of the fibrotic capsule was consistently reduced in devices with DEX compared to the control devices. Under light microscopy, we found the PI devices within the endoneurium of the nerve, surrounded by the fibrous capsule. There was no evidence of axonal degeneration that could be caused by the implantation (Figure S4, Supporting Information). This is in line with the results of functional tests performed during two months follow‐up in the rats of the two groups (see Figure S5, Supporting Information).

These findings demonstrate that covalent binding DEX to PI devices is effective for reducing FBR, similar to what has been reported with systemic DEX administration in rats.^[^ ^12^ ^]^ In this way, systemic side effects can be avoided, since only a small fraction of the drug is required compared to systemic administration. The use of DEX associated with a conductive polymer, such as PEDOT, has previously been shown to improve recording properties and reduce fibrotic‐associated tissue, enhancing the separation of neurons in brain electrode implants over time.^[^ ^13d^ ^]^ The coating of electrodes with PEDOT and its potential loading with DEX will complement the functionalization of the substrate in the future and allow full electrode functionality with respect to electrical stimulation.^[^ ^13d^ ^]^ In peripheral nerve interfaces, DEX was mixed with the silicon substrate in regenerative electrodes, but its release was not controlled.^[^ ^32^ ^]^ In that report, the author observed that DEX release resulted in a reduction in regenerating axon numbers after the nerve section, caused by the slowing in Wallerian degeneration.^[^ ^32^ ^]^ This is in contrast with our model in which longitudinal intraneural devices do not cause axonal damage and our findings that focal release of DEX did not have any deleterious effect in neither the nerve structure (see Figure 9; Figure S4, Supporting Information) nor function (Figure S5, Supporting Information). In intraneural interfaces, most of the device surface in contact with the nerve tissue is constituted by the substrate, in our case thin‐film PI. Therefore, the intended drug has to be strongly bound to the surface of the PI to enable a sustained release over the necessary period to effectively modulate the FRB.^[^ ^12^ ^]^

## 3 Conclusion

This study successfully demonstrated that the covalent surface functionalization of BPDA‐PDA with the corticosteroid drug dexamethasone represents an interesting and effective approach to mitigate FBR post‐implantation. The covalent bonding strategy developed in this study enabled a sustained release of DEX for 9 weeks, although traces of the drug can be still detected on the surface after a longer period. This has been shown to reduce both inflammatory responses and fibrotic encapsulation in vivo, as well as modulate macrophage activity in vitro. Furthermore, the modification at the surface level of the material did not significantly affect its mechanical properties, nor did it affect the biocompatibility if compared to the native material. Going more in‐depth, the in vitro tests confirmed that PI‐Si‐DEX substrates maintained neuronal viability and did not impair neurite growth, further supporting their biocompatibility. Moreover, the in vivo studies in rats showed that DEX‐bound polyimide devices led to significantly lower recruitment of inflammatory cells and thinner fibrotic capsules compared to non‐functionalized controls.

Overall, the covalent approach proposed in this study represents a promising strategy to improve the long‐term functionality not only of peripheral nerve interfaces but also of neural devices in general. By reducing the immune response and maintaining the biocompatibility of the material, this approach holds the potential for enhancing the performance and durability of implanted neural devices in clinical chronic applications.

In the next future, the proposed approach will be extended to a recording and/or stimulating neural implant, to evaluate the effect of the chemical strategy on the electrochemical properties of the microelectrodes.

## 4 Experimental Section

### Polyimide‐Based Specimens’ Fabrication

**Figure 10:**
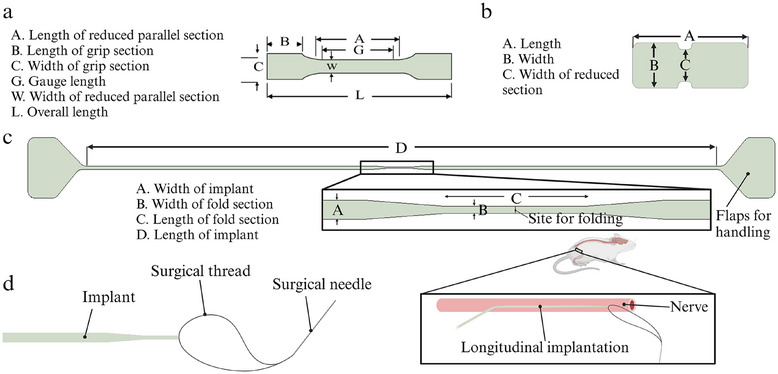
a) Tensile strength test specimen geometry and dimension based on ISO 527:2019 standard, with a modification to the grip length. Dimensions: A = 6 mm, B = 3.4 mm, C = 2 mm, G = 5 mm, L = 15 mm, W = 1 mm and with a device thickness of 4.46 ± 0.13 µm; b) in vitro test specimen with dimensions of A = 7.42 mm, B = 2.92 mm and C = 2.07 mm and with a device thickness of ≈13 µm; c) in vivo implant with A = 0.2 mm, B = 0.1 mm, C = 1.9 mm and D = 57 mm and with a thickness of 13 µm; d) method of longitudinal implantation into the sciatic nerve of the rat.

The PI samples were prepared by spin‐coating a layer of polyimide precursor (U‐Varnish S, UBE corporation) on a Si carrier. The spin parameters were a pre‐spinning speed of 500 rpm for 5 s, followed by a step of 3000 rpm for 30 s. After this, the samples were cured at 450 °C for 10 min, under a nitrogen atmosphere (YES‐PB6, Yield Engineering Systems Inc.), as depicted in Figure S6 (Supporting Information). The tensile strength test specimens (**Figure** **10**a) were fabricated with only one layer of PI while in the in vivo and in vitro test specimens (Figure 10b,c, respectively) another PI layer was deposited using the same procedure, with an oxygen plasma treatment step (30 s 100 W RIE, PlasmaTerm RIE) before the deposition. Photoresist (AZ 10 XT 520cP, Microchemicals) was deposited by spin coating and exposed using laser‐ (MLA 150, Heidelberg instruments) or photolithography (Suss MA6, SUSS Microtech) and subsequently etched with reactive ion etching (150 W RIE, 600 W ICP, Oxford PlasmaPro 100 ICP/RIE, Oxford instruments) to define the outline of the samples. A thickness value of 11.85 ± 0.06 mm was measured for the PI sample that was used for the chemical characterization as well as for the analysis of DEX release. This parameter was collected by means of a step D‐500 Profilometer (KLA Instruments, Milpitas, CA): data were recorded in a step‐up/down mode at a speed of 0.1 mm s^−1^ and a stylus force of 10.0 mg.

### Surface Functionalization

All chemicals and solvents were purchased by Merk and Carlo Erba Reagents, respectively, except otherwise specified.

### Preparation of PI‐CO_2_H Samples

PI pristine samples (≈2 cm^2^) were removed from silicon wafers and treated with a 5 m solution of potassium hydroxide (KOH) at 50° for 10 min. Then, the samples were removed from the alkaline solution and dipped in a 0.5 m hydrochloric acid (HCl) solution for 5 min at room temperature. A rinse with deionized water followed. After that, the samples were placed in a vial with deionized water at 37° for 18 h, to allow any impurities to be purged from the surface after the strong alkaline treatment.

### Preparation of PI‐OH Samples

PI‐CO_2_H samples (≈2 cm^2^) were placed in a vial and reacted with *Tris*(hydroxymethyl)aminomethane (Trizma base, 6 mg, 0.05 mmol) and *N, N′*‐Dicyclohexylcarbodiimide (DCC, 10.21 mg, 0.05 mmol) in absolute ethanol (3 mL) at 50° for 24 h. After the completion of the reaction, the samples were washed with methanol and dried.

### Preparation of PI‐Si Samples

PI‐OH (≈2 cm^2^) was treated in a toluene solution (4% *v*/*v*) of 3‐(Triethoxysylil) propylsuccinic anhydride (TESPSA, ABCR GmbH) for 4 days at room temperature. The silanized sample was washed with methanol several times and finally dried.

### Preparation of PI‐Si‐DEX Samples

PI‐Si samples (≈2 cm^2^) were placed in a vial with dexamethasone (DEX, 10 mg, 0.025 mmol, ABCR GmbH), DCC (5.6 mg, 0.025 mmol) and 4‐dimethylaminopyridine (DMAP, catalytic amount) in acetonitrile (3 mL) at 50° for 4 days. PI‐Si‐DEX samples were finally washed with methanol and dried.

### Attenuated Total Reflection Fourier Transform Infrared Spectroscopy (ATR‐FTIR)

ATR‐FTIR spectra were collected with a Nicolet iS50 spectrometer (Thermo Fisher Scientific) with a Ge‐ATR element fixed at a 45° incident angle. The penetration depth (*d_p_*) through the sample, defined as the distance at which the beam intensity was reduced to 1/e of its value at the interface, could be expressed by the formula:

(5) $$d_{p}=\frac{1}{2\pi \tilde{\nu }\sqrt{n_{0}^{2}sin^{2}\phi_{0}-n_{x}^{2}}}\;$$

where $\tilde{\nu }$ is the wavenumber (cm^−1^), *n*_0_ and ϕ_0_ are the refractive index and the angle of incidence of ATR crystal, respectively, while *n_x_* is the refractive index of the sample.^[^ ^38^ ^]^ In this study, a Ge‐ATR module was used, with a crystal refractive index value of 4 and an angle of incidence of 45°, according to the costumer. By employing a refractive index of 1.848 for PI, as reported in the literature, Equation (5) provides a penetration depth on the order of 419–437 nm at wavenumbers of 1770–1711 cm^−1^.^[^ ^16^ ^]^

### Surface Analysis

Contact angles between the surface and deionized water were measured and automatically calculated using an Ossila Contact Angle Goniometer: an average value was determined from five measurements.

Atomic force microscopy (AFM) images were collected using a Nanoscope III scanning probe microscope (Veeco Instruments). The instrument was equipped with a silicon tip (RTESP‐300 Bruker, spring constant 40 N m^−1^ and frequency resonance 300 kHz) and operated in tapping mode. The image was collected at a scan rate of 0.5 Hz, scan line of 512×256 pixels, and scan size of 5000 nm. Surface topographical analysis of raw AFM images was carried out with both NanoScope 1.5 and Gwyddion analysis software.

Scanning Electron Microscopy analysis (SEM) imaging was performed with a GEMINI SEM 460 ZEISS (EHT 5.00 kV, signal SE 2, probe 200 pA‐high vacuum). A layer of ≈5 nm of carbon was then deposited on all samples. Energy Dispersive X‐ray analysis (EDX) was collected with a Zeiss EVO 40 (working distance 9.5 mm, AZtec software).

### DEX Release Profile Analysis and Stability Test

The analysis of DEX release from PI‐Si‐DEX samples (≈2 cm^2^) was performed in a PBS solution (1 mL, 1 m, pH 7.4) at 37 °C. Analysis was conducted in triplicate. For each reading, the release solution was removed and stored at −20 °C. At the same time, to mimic circulating conditions characteristic of in vivo systems, after each sampling, the PBS supernatant in contact with PI‐Si‐DEX was replaced with fresh PBS solution. The sampling was carried out on days 1, 4, 7, 14, 21, 28, 35, 42, 49, and 63, which represent an overall period of 9 weeks. Evaluation of the release amount of DEX was performed by high‐pressure liquid chromatography (HPLC).

The stability of PI‐Si‐DEX was monitored by SEM‐EDX analysis. PI‐Si‐DEX was treated at 37 °C for 60 days in 0.1 m PBS at pH 7.4. Subsequently, an accelerated aging test (based on assumptions that only processes of first order occur) was performed at 57 °C for 15 days, corresponding to a further period of 60 days at 37 °C. The acceleration factor can be expressed according to Equation (6):

(6) $$t_{37^{\circ }}=t_{57^{\circ }}\;\times 2^{\frac{\Delta T}{10}}$$

where $t_{37^{\circ }}$ represents the equivalent aging time at 37 °C for a sample treated for a given amount of time at the temperature of 57 °C.^[^ ^39^ ^]^

### HPLC Analysis

Ultra‐pure water was obtained through a Milli‐Q‐ system (Millipore, USA), acetonitrile (ACN) and trifluoracetic acid (TFA) HPLC grade were purchased from Carlo Erba reagents (Milan, Italy). All release samples were stored at −20 °C until analysis. The samples were gradually brought to room temperature, diluted 1:1 with ACN, and vortexed for 5 min. Afterward, they were filtered using 0.22 µm nylon filters and injected into the HPLC system. HPLC separations were performed on a KNAUER AZURA HPLC system (KNAUER, Berlin, Germany) equipped with a quaternary low‐pressure gradient pump (max pressure: 862 bars), a column thermostat, an autosampler, and a photodiode array detector. The column used was a Phenomenex Luna Phenyl‐Hexyl 100 × 4.6 mm (L × I.D.) packed with 3 µm fully porous particles with 100 Å pore size. The mobile phases used were: A) Ultra‐pure water with 0.01% v/v TFA and B) ACN with 0.01% v/v TFA. The elution was performed in isocratic mode for 4 min at B = 40%, followed by a gradient to reach 90% B at minute 14, then maintained for 3 min. The initial mobile phase composition was then restored, and the column was allowed to re‐equilibrate for 3 min before proceeding with subsequent injections. The flow rate was set at 1 mL min^−1^, the injection volume was 30 µL, the column temperature was 30 °C and the detection wavelength was 242 nm. The retention time of DEX was ≈2.9 min. Quantification was performed through comparison with calibration curves, constructed within a range between 50 and 1.6 ppm. The limit of quantification (LOQ) and the limit of detection (LOD) measured by dilution of standard solutions were 0.26 and 0.13 µm, respectively.

### Tensile Test

The tensile testing of PI‐based substrates was conducted in accordance with ISO 527‐1:2019 standard. The tensile specimens featured a standardized hourglass geometry, with a gauge length of 5 mm, and a width of 1 mm. To prevent slippage, the grip length, originally smaller than ISO 527‐1 specifications, was doubled to 3.4 mm as it had been described in Figure 10a. The thickness of the PI specimens was measured using a Veeco Dektak 150 Profilometer, yielding an average thickness of 4.46 ± 0.13 µm. The test was performed using a DAGE 4000 Bond Tester equipped with a modular load cell WP‐10Kg. The equipment had a maximum load capacity of 10 N, and the crosshead speed was set to 10 µm s^−1^. A total of five samples were tested. Figure S7 (Supporting Information) provides a detailed illustration of the test setup.

### In Vitro Evaluation of Macrophage Response

Four‐week‐old female Sprague Dawley rats were euthanized with pentobarbital (200 mg kg^−1^ i.p.) and cleaned with ethanol 70%. The femur and tibia bones were dissected, bone epiphyses removed, and bone marrow flushed with chilled DPBS (Gibco, Cat #14190144). The collected extract was centrifuged at 1900 rpm for 10 min and cells were cultured in 100 mL Petri dishes with DMEMF/12 medium (Gibco, Cat #31330038) that contained 10% hiFBS (Gibco, Cat#A5256701) and 1X penicillin/streptomycin (Sigma, Cat #P0781). Macrophage colony‐stimulating factor (M‐CSF) (PrepoTech, Cat #400‐ 28) was added at 10 ng mL^−1^. Medium was replaced every three days. At 8 days in vitro, cells were reseeded in 24‐well plates at 3.5 × 10^5^ cells/well. In vitro test specimens (Figure 10b), PI‐OH, PI‐Si, or PI‐Si‐DEX were previously attached at the bottom of the plate. After 48 h, cells were treated for 3 h with LPS at a concentration of 10 µg mL^−1^ to induce a pro‐inflammatory reaction in the macrophages.

Production of NO by the reactive macrophages was measured in the supernatant using a modified Griess reaction. For this assay, 100 µL of each sample was incubated with 100 µL of Griess reagent (Sigma, Cat #G4410). After 15 min of incubation, the absorbance was measured at 540 nm using a Varikoskan Lux microplate reader (Thermo Scientific). The nitrite concentration was calculated by subtracting the absorbance of the sample from the blank, and the result was interpolated to the equation obtained from a standard curve of NaNO_2_ (Sigma, Cat #237213).

To measure the expression of several cytokines, RNA from the cultured macrophages was isolated using RNeasy Micro Kit (Qiagen, Cat #74004) and quantified using a NanoDrop. An amount of 30–100 ng of RNA was reverse transcribed into cDNA (Applied Biosystems, Cat# 4368813). The PCR reaction was conducted in 10 µL containing 1 µL cDNA template, 0.5 µL of each primer at 10 µm, and 5 µL of 2X Applied Biosystems Power SYBR Green PCR Mastermix (ThermoFisher Scientific) in nuclease‐free water. PCR was run on a CFX384 Touch Real‐Time PCR Detection System. The geometric mean of the expression levels of GAPDH was used to normalize the expression of Cp values. Primer sequences are listed in Table S10 (Supporting Information).

### In Vitro Evaluation of Neuronal Toxicity

The lumbar DRG were dissected from Sprague–Dawley rats, and placed in cold Gey's salt solution (Sigma, Cat #G9779) supplemented with 6 mg mL^−1^ glucose. DRG were cleaned and dissociated, first enzymatically in Hank's medium (Sigma, Cat #14170‐088) with 10% trypsin (Sigma, #T‐4674), 10% collagenase A (Sigma, Cat #C2674) and 10% DNAse (Roche, Cat #11284932001) for 40 min at 37 °C, followed by mechanical dissociation. Enzymes were inhibited with 10% hiFBS in DMEM (Sigma, Cat #41966052) and centrifuged at 900 rpm for 7 min. The supernatant was withdrawn, and 1 mL of culture medium was added, which consists of Neurobasal A, supplemented with 2% B27 (Gibco, Cat #17504044), 6 mg mL^−1^ glucose, 1 mm Glutamine (Gibco, Cat #35050‐038), 1X penicillin/streptomycin (Sigma, Cat #P0781). Plates were previously coated with 100 µg mL^−1^ poly‐D‐lysine (PDL, Sigma, Cat #P6407) and further coated with 1 µL mL^−1^ laminin (Sigma, Cat #L‐2020). The in vitro test specimens were attached at the bottom of the plate, where DRG neurons were cultured on top of control PI or PI‐Si‐DEX devices.

Cell viability was assessed by counting the number of neurons in the same fields 1 and 3 days after seeding under a phase‐contrast microscope (Olympus CKS41). Viable neurons were identified by a bright outline that is absent in dead cells. In addition, the MTT reduction assay was performed in the same cultures. Other cell cultures, under the same conditions, were fixed with 4% paraformaldehyde for 20 min, and neurons were immunostained with mouse primary antibody against ß‐III‐tubuline (1:500, Biolegend, Cat #MMS435P) and DAPI (1:1000, Sigma, Cat #D9564‐10MG). The length of the longest neurite from each ß‐III‐tubulin‐positive neuron present in each well of the culture plates was measured, with this measurement performed separately for each experimental condition. Three experimental conditions were established for each culture plate: control, PI, and PI‐Si‐DEX, and three different cultures were analyzed for each condition. The measurements were taken using an epifluorescence microscope (Nikon ECLIPSE Ni) attached to a digital camera (DS‐Ri2) and analyzed with ImageJ software. The average of the obtained neurite lengths was then calculated to represent the mean value at each experimental point.

### In Vivo Assay

Operations were performed under anesthesia with ketamine/xylazine (90/10 mg kg^−1^ i.p.) on female Sprague Dawley rats. In the study, a total of 20 animals were used, divided into two subsets evaluated at 2 weeks (2w) and 8 weeks (8w) after implantation. At each time point, two groups of 5 rats were established: a control group and a treatment group. The control group was implanted with a PI‐only device, while the treatment group was implanted with a PI‐Si‐DEX device. Only one device was implanted per animal, with the contralateral leg remaining intact and used as a normal control reference. The sciatic nerve was surgically exposed to the midthigh and carefully freed from adherence to surrounding tissues. The test specimens (Figure 10c), following the design of longitudinal intrafascicular electrodes (LIFE), were inserted longitudinally in the tibial fascicle of the sciatic nerve with the help of a straight needle attached to a 10–0 loop thread (STC–6, Ethicon), as shown in Figure 10d.^[^ ^4a^ ^]^ After surgery, all animals were housed under standard conditions. Animal experiments were performed following protocols approved by the Ethical Committee of the Universitat Autònoma de Barcelona (procedure CEEAH‐5024 to XN), in accordance with the European Communities Council Directive 2010/63/EU. The ARRIVE guidelines were followed in the experimental work, and adequate measures were taken to minimize pain and discomfort during surgery and postoperative follow‐up.

After 2 weeks and 8 weeks, half of the animals were euthanized and perfused with 4% paraformaldehyde in 0.1 m phosphate buffer (PB). The sciatic nerves where the devices had been implanted were dissected, cryoprotected in 30% sucrose, and cut into 15‐µm‐thick transversal sections using a cryostat (Leica CM190, Leica Microsystems). After thawing and blocking with normal donkey serum, slides were incubated with primary antibodies for Iba1 (rabbit; 1:500; Wako) to label infiltrating macrophages and for neurofilament 200 kDa clone RT97 (mouse; 1:200; Developmental Studies Hybridoma Bank) to label axons. Slides were washed and incubated with AlexaFluor 488 donkey anti‐mouse and AlexaFluor 555 donkey anti‐rabbit secondary antibodies (Invitrogen). Finally, slides were mounted with Fluoromont containing DAPI (Sigma).

To study implant‐associated FBR, for both the quantification of Iba1⁺ cells and the measurement of capsule thickness, five different transverse sections of the sciatic nerve were analyzed per animal, selected along its length with a minimum spacing of 250 µm between them. For macrophage counting, the cell density was assessed by quantifying the total number of Iba1^+^ cells present within the entire area of each nerve cross‐section, using a custom‐made macro for Image J software. Regarding capsule thickness measurement, the distance between the surface of the polyimide implant and the surrounding tissue was quantified on both sides of the implant. This area was then divided by the width of the polyimide strip, yielding a relative capsule thickness index that enables normalized comparison between samples.

Another segment of the implanted nerves was processed for light microscopy, by fixation in 3% glutaraldehyde and 3% paraformaldehyde, and then in 2% OsO_4_. These segments were embedded in epon resin, 0.5 µm thin sections cut with an ultramicrotome, and stained with toluidine blue. The sections were viewed under a microscope for general imaging and for detection of possible axonal degeneration.

### Statistical Analysis

The in vitro and in vivo data are presented as mean ± SD. Statistical comparisons were made using the appropriate test, two‐way ANOVA followed by the Bonferroni post‐hoc test for normal data or Kruskal–Wallis tests for non‐normal distributed data. The test was applied in each case and the sample size is indicated in the figure captions. Differences were considered significant when *p* < 0.05. The GraphPad Prism 8 software was used for analyses.

The tensile test data are presented as mean ± SD. Statistical comparisons were performed using a one‐way ANOVA for normally distributed data. The specific test used in each case, along with the sample size, is indicated in the figure captions. Differences were considered statistically significant at *p* < 0.05. All analyses were conducted using Origin software.

## Conflict of Interest

The authors declare no conflict of interest.

## Supporting information



Supporting Information

## Data Availability

The data that support the findings of this study are available from the corresponding author upon reasonable request.

## References

[1] P. S. Olofsson , C. Bouton , J. Intern. Med. 2019, 286, 237. 31429132 10.1111/joim.12967

[2] X. Navarro , T. B. Krueger , N. Lago , S. Micera , T. Stieglitz , P. Dario , J. Peripher. Nerv. Syst. 2005, 10, 229. 16221284 10.1111/j.1085-9489.2005.10303.x

[3] a) D. Shahriari , D. Rosenfeld , P. Anikeeva , Neuron 2020, 108, 270; 33120023 10.1016/j.neuron.2020.09.025

[4] a) N. de la Oliva , X. Navarro , J. Del Valle , J. Biomed. Mater. Res., Part A 2018, 106, 746; 10.1002/jbm.a.36274 29052368

[5] T. Stieglitz , H. r. Beutel , M. Schuettler , J. U. Meyer , Biomed. Microdevices 2000, 2, 283.

[6] C. Hassler , T. Boretius , T. Stieglitz , J. Polym. Sci., Part B: Polym. Phys. 2010, 49, 18.

[7] a) B. Rubehn , T. Stieglitz , Biomaterials 2010, 31, 3449; 20144477 10.1016/j.biomaterials.2010.01.053

[8] a) T. Boretius , J. Badia , A. Pascual‐Font , M. Schuettler , X. Navarro , K. Yoshida , T. Stieglitz , Biosens. Bioelectron. 2010, 26, 62; 20627510 10.1016/j.bios.2010.05.010

[9] P. Čvančara , G. Valle , M. Müller , I. Bartels , T. Guiho , A. Hiairrassary , F. Petrini , S. Raspopovic , I. Strauss , G. Granata , E. Fernandez , P. M. Rossini , M. Barbaro , K. Yoshida , W. Jensen , J.‐L. Divoux , D. Guiraud , S. Micera , T. Stieglitz , npj Flexible Electron. 2023, 7, 51.

[10] a) M. B. Christensen , S. M. Pearce , N. M. Ledbetter , D. J. Warren , G. A. Clark , P. A. Tresco , Acta Biomater. 2014, 10, 4650; 25042798 10.1016/j.actbio.2014.07.010

[11] N. de la Oliva , M. Mueller , T. Stieglitz , X. Navarro , J. Del Valle , Sci. Rep. 2018, 8, 5965. 29654317 10.1038/s41598-018-24502-z PMC5899141

[12] N. De la Oliva , X. Navarro , J. Del Valle , Anat. Rec. 2018, 301, 1722. 10.1002/ar.23920 30353712

[13] a) S. Carli , C. Trapella , A. Armirotti , A. Fantinati , G. Ottonello , A. Scarpellini , M. Prato , L. Fadiga , D. Ricci , Chem.–A Eur. J. 2018, 24, 10300; 10.1002/chem.201801499 29799647

[14] C. Liu , M. A. Nguyen , A. Alvarez‐Ciara , M. Franklin , C. Bennett , J. B. Domena , N. C. Kleinhenz , G. A. Blanco Colmenares , S. Duque , A. F. Chebbi , B. Bernard , J. H. Olivier , A. Prasad , ACS Appl. Bio Mater. 2020, 3, 4613. 10.1021/acsabm.0c00506 35025460

[15] a) E. Melnik , R. Bruck , R. Hainberger , M. Lammerhofer , Anal. Chim. Acta 2011, 699, 206; 21704776 10.1016/j.aca.2011.05.017

[16] K. W. Lee , S. P. Kowalczyk , J. M. Shaw , Langmuir 2002, 7, 2450.

[17] H. Okumura , T. Takahagi , N. Nagai , S. Shingubara , J. Polym. Sci., Part B: Polym. Phys. 2003, 41, 2071.

[18] a) C. R. Nuttelman , M. C. Tripodi , K. S. Anseth , J. Biomed. Mater. Res. 2006, 76, 183; 10.1002/jbm.a.30537 16265650

[19] B. Neises , W. Steglich , Angew. Chem. Int. Ed. Engl. 2003, 17, 522.

[20] a) A. Y. Fadeev , T. J. McCarthy , Langmuir 2000, 16, 7268;

[21] a) G. Zhou , H. Loppnow , T. Groth , Acta Biomater. 2015, 26, 54; 26292266 10.1016/j.actbio.2015.08.020

[22] a) T. Scherzer , Vib. Spectrosc. 2002, 29, 139;

[23] B. H. Stuart , in Infrared Spectroscopy: Fundamentals and Applications, John Wiley & Sons, Ltd, Hoboken, NJ, USA, 2004.

[24] T. Verdianz , H. Simbürger , R. Liska , Eur. Polym. J. 2006, 42, 638.

[25] F. Ahangaran , A. H. Navarchian , Adv. Colloid Interface Sci. 2020, 286, 102298. 33171357 10.1016/j.cis.2020.102298

[26] W. Yoshida , R. P. Castro , J.‐D. Jou , Y. Cohen , Langmuir 2001, 17, 5882.

[27] A. I. Barabanova , T. A. Pryakhina , E. S. Afanas'ev , B. G. Zavin , Y. S. Vygodskii , A. A. Askadskii , O. E. Philippova , A. R. Khokhlov , Appl. Surf. Sci. 2012, 258, 3168.

[28] a) M. L. Bruschi , in Strategies to Modify the Drug Release from Pharmaceutical Systems, Woodhead Publishing, Cambridge, UK, 2015;

[29] a) J. Yao , S. Zhang , W. Li , Z. Du , Y. Li , RSC Adv. 2016, 6, 515;

[30] Y. Zhong , R. V. Bellamkonda , Brain Res. 2007, 1148, 15. 17376408 10.1016/j.brainres.2007.02.024 PMC1950487

[31] C. L. Cope , E. Black , Br. Med. J. 1958, 1, 1020. 13536407 10.1136/bmj.1.5078.1020 PMC2028647

[32] J. J. FitzGerald , J. Neural Eng. 2016, 13, 026006. 26824180 10.1088/1741-2560/13/2/026006

[33] a) D. Y. Vogel , J. E. Glim , A. W. Stavenuiter , M. Breur , P. Heijnen , S. Amor , C. D. Dijkstra , R. H. Beelen , Immunobiology 2014, 219, 695; 24916404 10.1016/j.imbio.2014.05.002

[34] X. Huang , Y. Li , M. Fu , H. B. Xin , Methods Mol. Biol. 2018, 1784, 119. 29761394 10.1007/978-1-4939-7837-3_12 PMC8875934

[35] a) C. Bogdan , Nat. Immunol. 2001, 2, 907; 11577346 10.1038/ni1001-907

[36] B. Rodriguez‐Meana , J. Del Valle , D. Viana , S. T. Walston , N. Ria , E. Masvidal‐Codina , J. A. Garrido , X. Navarro , Adv. Sci. 2024, 11, 2308689. 10.1002/advs.202308689 PMC11304253 38863325

[37] a) P. Lamoureux , J. Zheng , R. E. Buxbaum , S. R. Heidemann , J. Cell Biol. 1992, 118, 655; 1639849 10.1083/jcb.118.3.655 PMC2289549

[38] V. P. Tolstoy , I. V. Chernyshova , V. A. Skryshevsky , in Handbook of Infrared Spectroscopy of Ultrathin Films, Wiley Press, Hoboken, NJ, USA, 2003.

[39] B. J. Lambert , F.‐W. Tang , Radiat. Phys. Chem. 2000, 57, 349.

