# Supplementary material for: Covalent Binding of Dexamethasone to Polyimide Improves Biocompatibility of Neural Implantable Devices
Source: Adv Healthc Mater. 2025 Jun 17;14(21):2405004. doi: 10.1002/adhm.202405004 (PMC12365622; doi:10.1002/adhm.202405004)
Supplement: Supplementary file 1 — Supporting Information [file ADHM-14-0-s001.docx]

Supporting Information

Covalent Binding of Dexamethasone to Polyimide Improves Biocompatibility of Neural Implantable Devices

Giulia Turrin, Jose Crugeiras, Chiara Bisquoli, Davide Barboni, Martina Catani, Bruno Rodríguez-Meana, Rita Boaretto, Michele Albicini, Stefano Caramori, Claudio Trapella, Thomas Stieglitz, Yara Baslan, Hanna Karlsson-Fernberg, Fernanda L. Narvaez-Chicaiza, Edoardo Marchini, Alberto Cavazzini, Ruben López-Vales, Maria Asplund, Xavier Navarro, Stefano Carli*


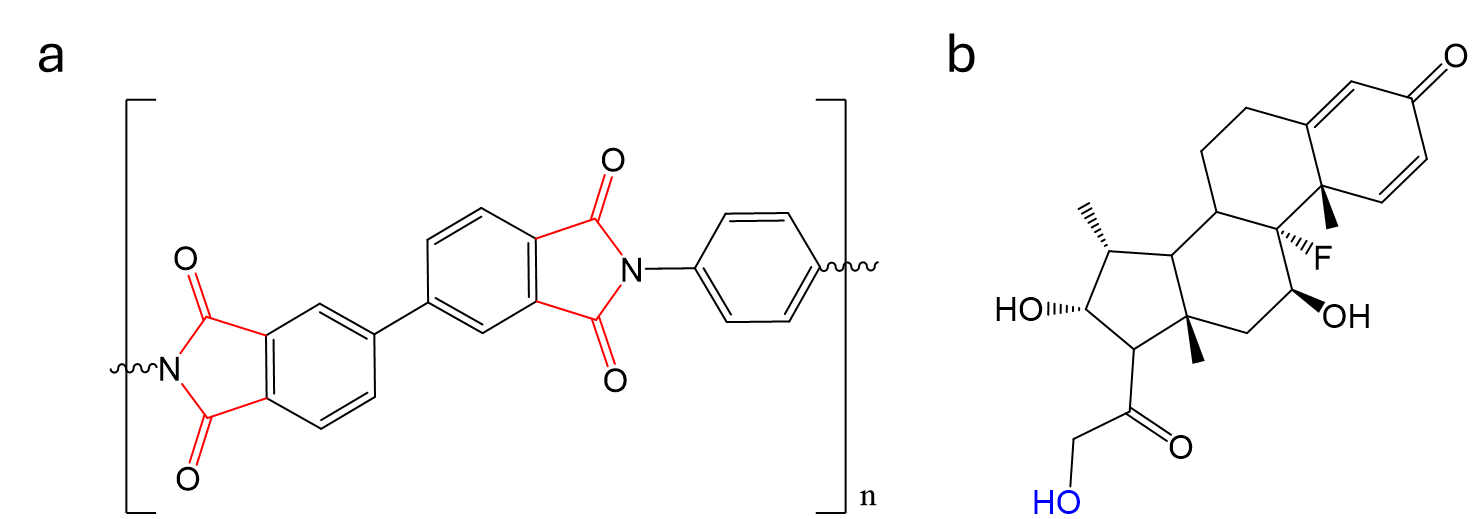


**Figure S1**. Chemical structure of a) PI and b) DEX; the imide ring of PI and the primary OH site of DEX is reported in red or blue, respectively.

**Table S1**. Estimation of the penetration depth for the KOH treatment to give PI-CO_2_H (data are reported as mean values ± SD, n = 4): comparison between ATR-FTIR and FTIR (transmission mode) methods.

|  | **PI** | **PI-CO_2_H** | **Method** |
| --- | --- | --- | --- |
| $A_{PI}$ | 0.293 ± 0.003 | // | ATR-FTIR |
| $A_{PI^{'}}$ | // | 0.16 ± 0.02 |  |
| $t_{PI^{'}}$ (μm) | // | 0.23 ± 0.02 |  |
| $t_{CO_{2}H}$(μm) | // | 0.10 ± 0.01 |  |
| $A_{PI}$ | 0.0585 ± 0.0005 | // | FTIR |
| $A_{PI^{'}}$ | // | 0.029 ± 0.004 |  |
| $t_{PI^{'}}$ (μm) | // | 0.21 ± 0.03 |  |
| $t_{CO_{2}H}$(μm) | // | 0.10 ± 0.02 |  |

**Table S2**. Thickness measurements (data are reported as mean values ± SD).

|  | **PI** | **PI-CO_2_H** |
| --- | --- | --- |
| **thickness (μm)** | 11.85 ± 0.06 | 11.8 ± 0.1 |

**Table S3**. Contact angle measurements (data are reported as mean values ± SD, n = 4).

|  | θ  *degrees* |
| --- | --- |
| **PI** | 66 ± 4 |
| **PI-CO_2_H** | 53 ± 4 |
| **PI-OH** | 57 ± 4 |
| **PI-Si** | 57 ± 3 |
| **PI-Si-DEX** | 53 ± 8 |

**Table S4**. Quantitative AFM analysis of PI and PI-Si-DEX.

|  | ***R_q_***  *nm* | ***R_a_***  *nm* | ***R_max_***  *nm* |
| --- | --- | --- | --- |
| ***PI*** | *0.63* | *0.487* | *15.6* |
| ***PI-Si-DEX*** | *26.4* | *21.1* | *185* |


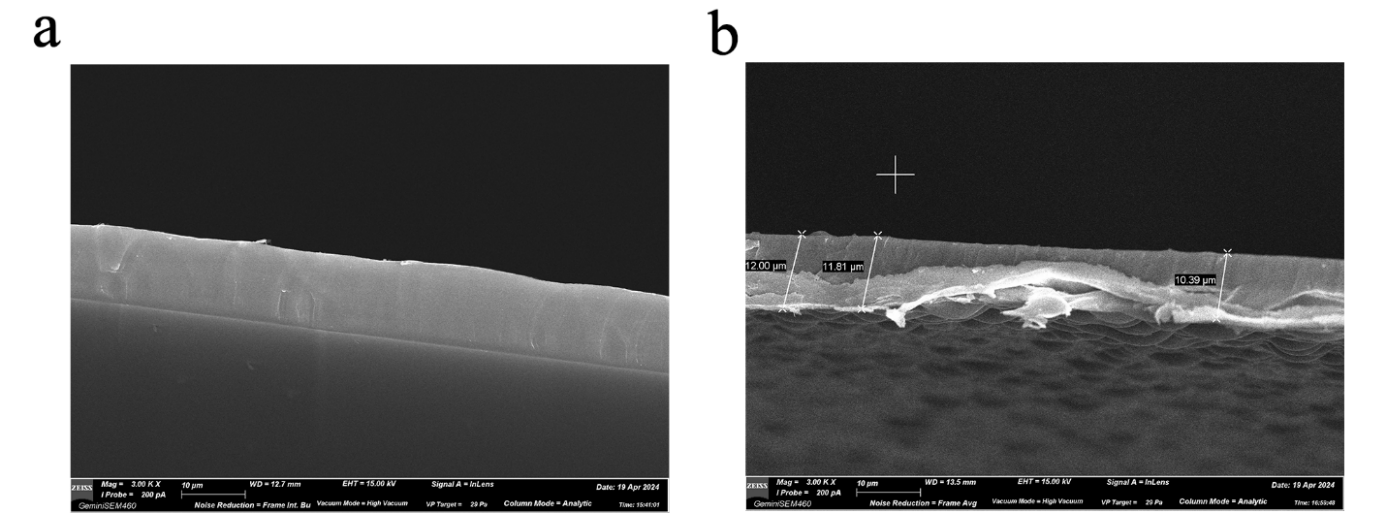


**Figure S2**. Cross-sectional SEM analysis of a) PI and b) PI-Si-DEX.

**Table S5**. Experimental single release data for three samples of PI-Si-DEX

| **Day** | **single release**  **[****nmol cm^-2^]** | | | **single release**  **[μmol L^-1^]** | | |
| --- | --- | --- | --- | --- | --- | --- |
|  | **Sample 1** | **Sample 2** | **Sample 3** | **Sample 1** | **Sample 2** | **Sample 3** |
| 1 | 19.1 | 9.3 | 5.7 | 47.6 | 21.2 | 20.1 |
| 4 | 25.6 | 12.7 | 8.9 | 63.8 | 29.1 | 31.3 |
| 7 | 8.9 | 6.2 | 2.9 | 22.2 | 14.1 | 10.2 |
| 14 | 3.2 | 3.7 | 4 | 7.9 | 8.5 | 14.0 |
| 21 | 1.8 | 2.4 | 2.1 | 4.5 | 5.6 | 7.2 |
| 28 | 1.3 | 1.4 | 0.57 | 3.1 | 3.2 | 2.0 |
| 35 | 0.69 | 2.1 | 0.47 | 1.8 | 5.1 | 1.6 |
| 42 | 0.37 | 0.87 | 0.34 | 0.95 | 2.0 | 1.2 |
| 49 | 0.23 | 0.56 | 0.31 | 0.6 | 1.3 | 1.1 |
| 63 | 0.19 | 0.72 | 0.23 | 0.48 | 1.7 | 0.8 |

**Table S6**. Experimental cumulative release data for three samples of PI-Si-DEX

| **Day** | **cumulative release**  **[nmol cm^-2^]** | | |
| --- | --- | --- | --- |
|  | **Sample 1** | **Sample 2** | **Sample 3** |
| 1 | 19.1 | 9.3 | 5.7 |
| 4 | 44.8 | 22.0 | 14.7 |
| 7 | 53.7 | 28.2 | 17.6 |
| 14 | 56.9 | 31.9 | 21.6 |
| 21 | 58.7 | 34.3 | 23.7 |
| 28 | 60.0 | 35.7 | 24.2 |
| 35 | 60.7 | 37.9 | 24.7 |
| 42 | 61.0 | 39.8 | 25.1 |
| 49 | 61.3 | 39.3 | 25.4 |
| 63 | 61.5 | 40.1 | 25.6 |

**Table S7**. Relevant parameters obtained by fitting release data of DEX release (data are reported as mean values ± SD).

| **model** | **Sample**  **R^2^** | | |
| --- | --- | --- | --- |
|  | **1** | **2** | **3** |
| Zero order | 0.669 | 0.819 | 0.782 |
| First order | 0.918 | 0.974 | 0.979 |
| Higuchi | 0.797 | 0.917 | 0.894 |
| Korsmeyer-Peppas | 0.674 | 0.845 | 0.787 |
| Weibull | 0.792 | 0.906 | 0.839 |
| Biexponential | 0.999 | 0.999 | 0.999 |

**Table S8**. Analysis for DEX release: kinetic model parameters obtained by fitting the experimental data with equation (4) of the manuscript (data are reported as mean values ± SD).

| ***Sample*** | ***A*** | ***k_1_***  ***day^-1^*** | ***t_1_***  ***day*** | ***B*** | ***k_2_***  ***day^-1^*** | ***t_2_***  ***day*** | ***D_Total_***  ***nmol cm^-2^*** |
| --- | --- | --- | --- | --- | --- | --- | --- |
| 1 | -53 ± 2 | 0.42 ± 0.04 | 2.3 ± 0.2 | -9 ± 2 | 0.05 ± 0.02 | 20 ± 8 | 62 ± 1 |
| 2 | -25 ± 2 | 0.37 ± 0.07 | 2.7 ± 0.5 | -16 ± 2 | 0.04 ± 0.01 | 27 ± 8 | 42 ± 1 |
| 3 | -16 ± 1 | 0.31 ± 0.04 | 3.2 ± 0.4 | -9 ± 1 | 0.06 ± 0.01 | 18 ± 1 | 26 ± 0.1 |

**Table S9**. Theoretical single release data from fitting parameters reported in Table S8 (data are reported as mean values ± SD).

| **Day** | **DEX release**  ***nmol cm^-2^*** | **Day** | **DEX release**  ***nmol cm^-2^*** | **Day** | **DEX release**  ***nmol cm^-2^*** | **Day** | **DEX release**  ***nmol cm^-2^*** |
| --- | --- | --- | --- | --- | --- | --- | --- |
| 1 | 11 ± 7 | 16 | 0.29 ± 0.07 | 31 | 0.12 ± 0.06 | 46 | 0.06 ± 0.04 |
| 2 | 7 ± 5 | 17 | 0.27 ± 0.07 | 32 | 0.12 ± 0.05 | 47 | 0.06 ± 0.04 |
| 3 | 5 ± 3 | 18 | 0.25 ± 0.07 | 33 | 0.11 ± 0.05 | 48 | 0.06 ± 0.03 |
| 4 | 4 ± 2 | 19 | 0.23 ± 0.07 | 34 | 0.11 ± 0.05 | 49 | 0.05 ± 0.03 |
| 5 | 3 ± 1 | 20 | 0.22 ± 0.07 | 35 | 0.10 ± 0.05 | 50 | 0.05 ± 0.03 |
| 6 | 1.9 ± 0.6 | 21 | 0.21 ± 0.07 | 36 | 0.10 ± 0.05 | 51 | 0.05 ± 0.03 |
| 7 | 1.4 ± 0.4 | 22 | 0.20 ± 0.07 | 37 | 0.09 ± 0.05 | 52 | 0.05 ± 0.03 |
| 8 | 1.1 ± 0.2 | 23 | 0.19 ± 0.07 | 38 | 0.09 ± 0.05 | 53 | 0.04 ± 0.03 |
| 9 | 0.8 ± 0.1 | 24 | 0.18 ± 0.07 | 39 | 0.09 ± 0.05 | 54 | 0.04 ± 0.03 |
| 10 | 0.66 ± 0.08 | 25 | 0.17 ± 0.06 | 40 | 0.08 ± 0.04 | 55 | 0.04 ± 0.03 |
| 11 | 0.54 ± 0.07 | 26 | 0.16 ± 0.06 | 41 | 0.08 ± 0.04 | 56 | 0.04 ± 0.03 |
| 12 | 0.46 ± 0.07 | 27 | 0.15 ± 0.06 | 42 | 0.07 ± 0.04 | 57 | 0.04 ± 0.03 |
| 13 | 0.40 ± 0.07 | 28 | 0.14 ± 0.06 | 43 | 0.07 ± 0.04 | 58 | 0.04 ± 0.03 |
| 14 | 0.35 ± 0.07 | 29 | 0.14 ± 0.06 | 44 | 0.07 ± 0.04 | 59 | 0.03 ± 0.02 |
| 15 | 0.32 ± 0.07 | 30 | 0.13 ± 0.06 | 45 | 0.06 ± 0.04 | 60 | 0.03 ± 0.02 |


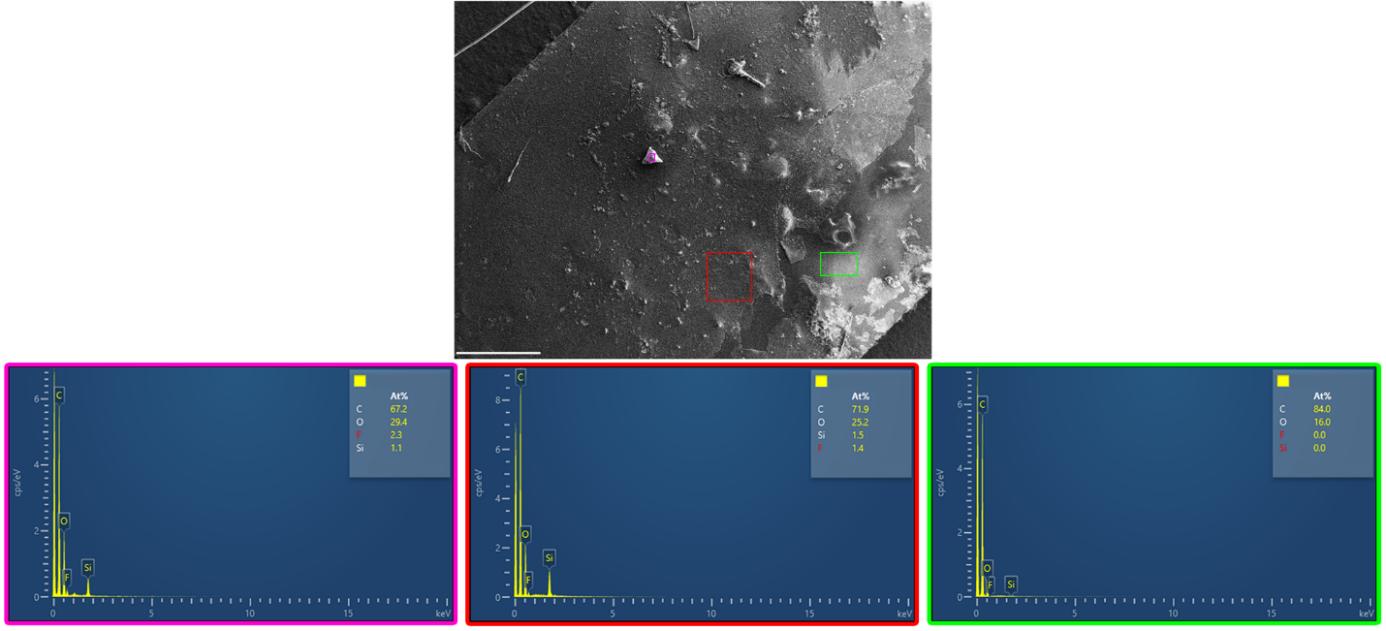
 **Figure S3**. SEM/EDX elemental analysis of PI-Si-DEX for C, O, Si and F atoms. In the upper panel the three analysed areas are indicated by the purple, red and green rectangles, respectively, and their experimental data (atomic %) are reported in the lower panel, accordingly. Scale bar is 1 mm.


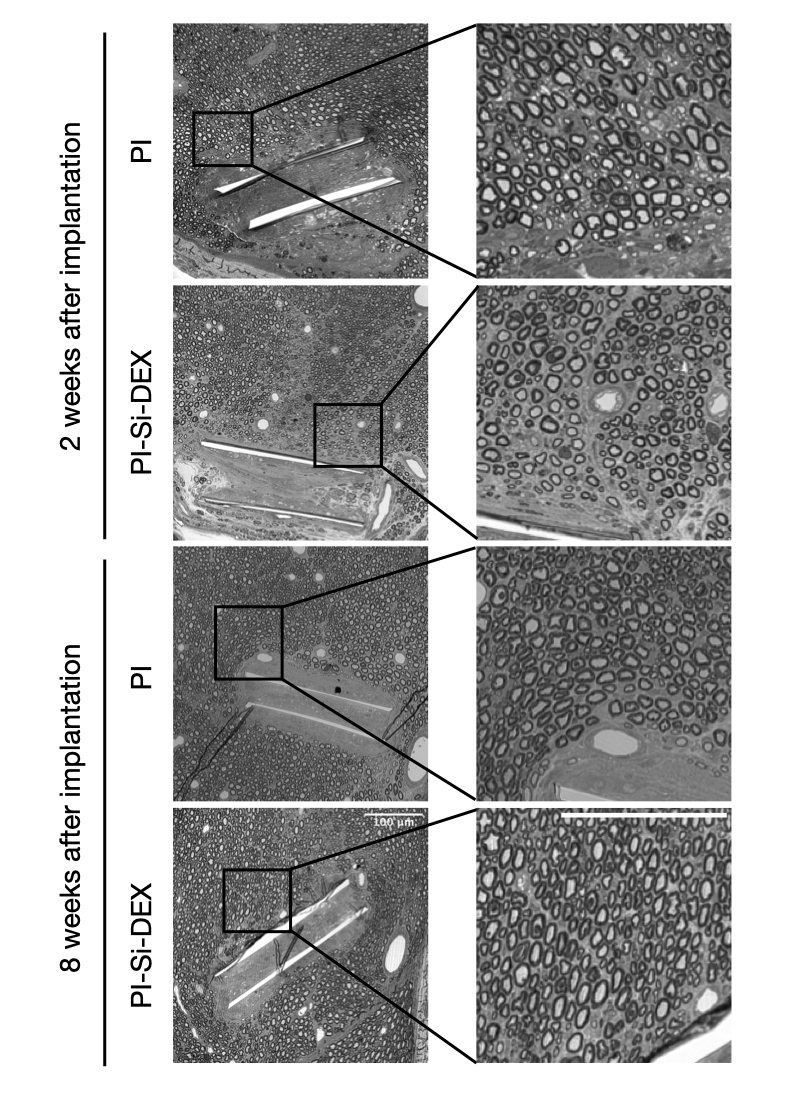


**Figure S4.** Representative images taken under light microscopy (LM) of transverse sections of the sciatic nerve implanted with intraneural PI control and PI loaded with dexamethasone (PI-Si-DEX) devices. In the left column the same micrographs included in Figure 9 are shown at 40x magnification, whereas in the right column insets are shown at 100x. Note the connective capsule around the PI devices, and the normal microstructure of the endoneurium, without any evidence of axonal damage. Scale bars: 100 µm.

**Figure S5.** Results of the neurophysiological assessment in rats with a PI control device (PI) or a PI-Si-DEX device implanted in the tibial nerve during 2 and 8 weeks (for methods see references 4a, 11). a: Algesimetry test results expressed as percentage of force threshold for withdrawal when applying a mechanical probe vs. the contralateral control paw of animals before the implantation (Pre) and after the implantation. No differences were found in the implanted hindlimb compared to the intact hindlimb. b: Walking track test results showing the plot of the Sciatic Functional Index obtained comparing the implanted and the contralateral paw. Values ​​near 0 indicate a normal pattern of locomotion. Motor nerve conduction test results of animals after implantation of PI devices for 2 and 8 weeks. c: CMAP amplitude of plantar interossei and gastrocnemius muscles. d: CMAP onset latency of the same muscles. There were similar values ​​of nerve conduction in implanted nerves as in the contralateral control values. No significant differences were found between groups with PI control or PI-Si-DEX device implanted for any of the tests. Two-way ANOVA followed by Bonferroni post-hoc test.


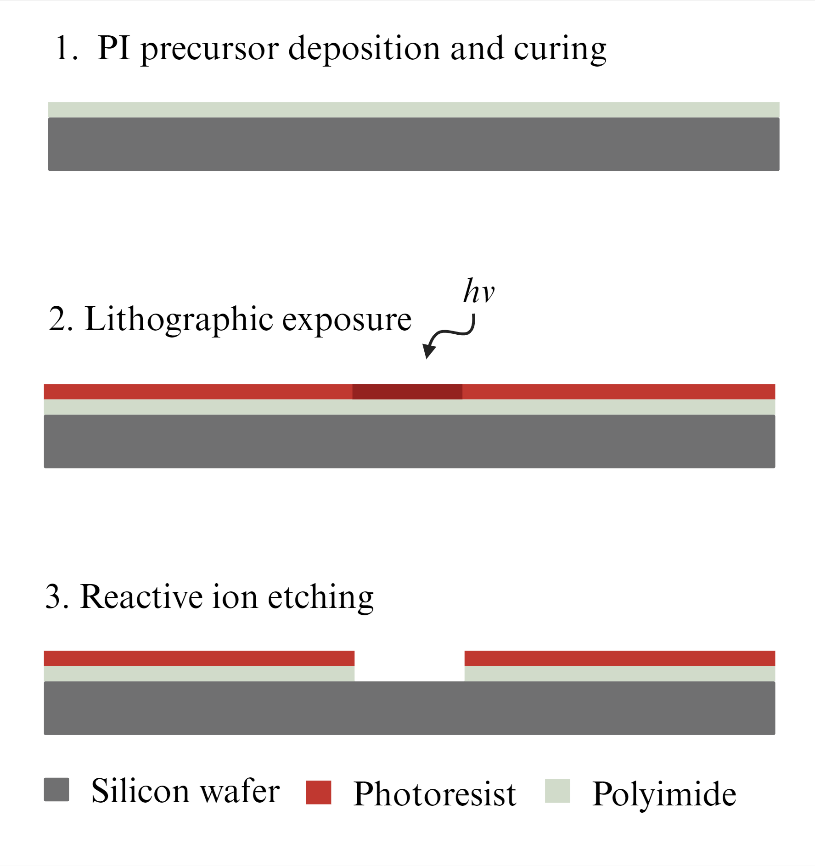


**Figure S6**. Microfabrication process of test specimens made from BPDA-PDA polyimide film.


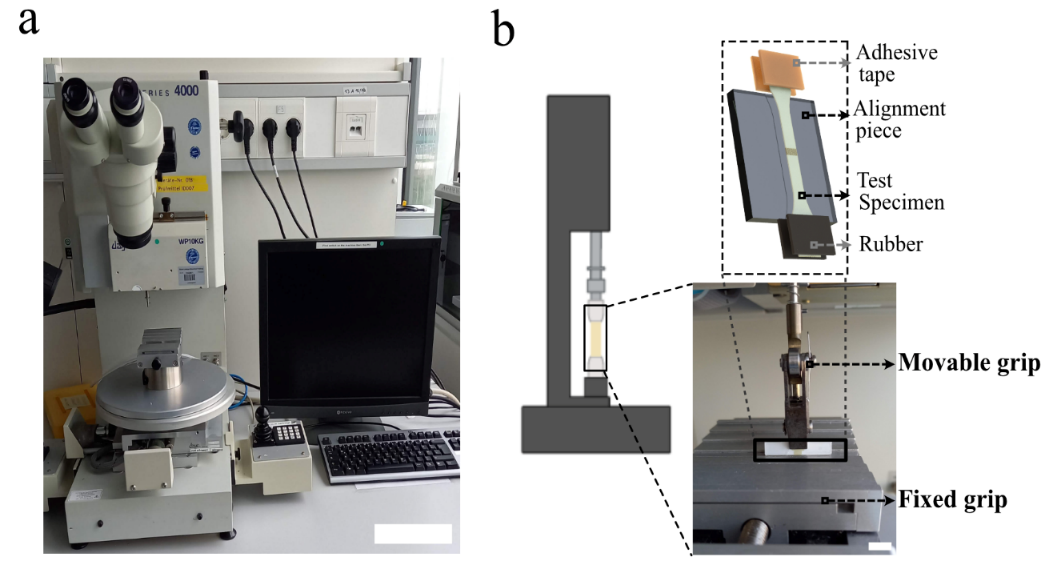


**Figure S7.** Tensile test: (a) DAGE 4000 bond tester used for tensile measurements, equipped with a WP10KG cartridge for destructive pull testing. (b) Sample mounting on the bond tester for tensile testing. The setup included an acrylic alignment piece to position the specimen both vertically and horizontally, adhesive tape to enhance grip force while fitting within the movable grip’s thickness tolerance, and rubber on the fixed grip to prevent slippage and reduce risk of premature fracture from excessive pressure on the specimen.

**Table S10**: List of primers used for the determination of cytokines mRNA.

|  | | |
| --- | --- | --- |
| **GENE** | **DIRECTION** | **SEQUENCE** |
| GAPDH | Reverse | 5′-TCCGTTGTCATACCAGGAAAT-3′ |
|  | Forward | 5′-GGTCATCCCTGAGCTGAAC G-3′ |
| IL-6 | Reverse | 5’- TTGGATGGTCTTGGTCCTTAGCC-3′ |
|  | Forward | 5’- CCTACCCCAACTTCCAATGCTC-3′ |
| IL-1ß | Reverse | 5’- CACCTCTCAAGCAGAGCACAG -3′ |
|  | Forward | 5’- GGGTTCCATGGTGAAGTCAAC-3′ |
| IL-10 | Reverse | 5’- GGGGAGAAATCGATGACAGC-3′ |
|  | Forward | 5’- GCAGGACTTTAAGGGTTACTTGG-3’ |
